# DNAi: an open-source AI tool for unbiased DNA fiber analysis

**DOI:** 10.1093/nar/gkag335

**Published:** 2026-04-17

**Authors:** Clément Playout, Yosra Mehrjoo, Renaud Duval, Marie Carole Boucher, Santiago Costantino, Hugo Wurtele

**Affiliations:** Research Center, Maisonneuve-Rosemont Hospital, 5415, boulevard de l’Assomption Montréal, H1T 2M4 Québec, Canada; Department of Ophthalmology, Université de Montréal, 2900 Edouard-Montpetit, H3T 1J4 Québec, Canada; Research Center, Maisonneuve-Rosemont Hospital, 5415, boulevard de l’Assomption Montréal, H1T 2M4 Québec, Canada; Molecular Biology Program, Université de Montréal, 2900 Edouard-Montpetit, H3T 1J4 Québec, Canada; Research Center, Maisonneuve-Rosemont Hospital, 5415, boulevard de l’Assomption Montréal, H1T 2M4 Québec, Canada; Department of Ophthalmology, Université de Montréal, 2900 Edouard-Montpetit, H3T 1J4 Québec, Canada; Research Center, Maisonneuve-Rosemont Hospital, 5415, boulevard de l’Assomption Montréal, H1T 2M4 Québec, Canada; Department of Ophthalmology, Université de Montréal, 2900 Edouard-Montpetit, H3T 1J4 Québec, Canada; Research Center, Maisonneuve-Rosemont Hospital, 5415, boulevard de l’Assomption Montréal, H1T 2M4 Québec, Canada; Department of Ophthalmology, Université de Montréal, 2900 Edouard-Montpetit, H3T 1J4 Québec, Canada; Research Center, Maisonneuve-Rosemont Hospital, 5415, boulevard de l’Assomption Montréal, H1T 2M4 Québec, Canada; Department of Medicine, Université de Montréal, 2900 Edouard-Montpetit, H3T 1J4 Québec, Canada

## Abstract

DNA fiber assays are powerful tools for investigating replication dynamics at the single-molecule level. However, their application and widespread adoption has been hampered by the labor-intensive and tedious nature of manual analysis of large numbers of images. Quantification of labeled DNA fibers typically depends on subjective examination, selection, and annotation of individual fibers from fluorescence microscopy images reducing inter-user consistency, reproducibility, and experimental throughput. To address these issues, we developed DNAi, a computer vision tool based on deep learning allowing automated detection and quantification of labeled DNA fiber length. DNAi was trained on a large and diverse dataset of manually annotated images of DNA fibers and matches human performance and accuracy in segmentation and length measurement across a wide range of experimental conditions. The open-source tool includes a user-friendly interface, which permits visual validation and manual selection of segmented fibers. Overall, DNAi enables robust, rapid, and reproducible DNA fiber analysis, and is freely available.

## Graphical Abstract

**Graphical Abstract:**
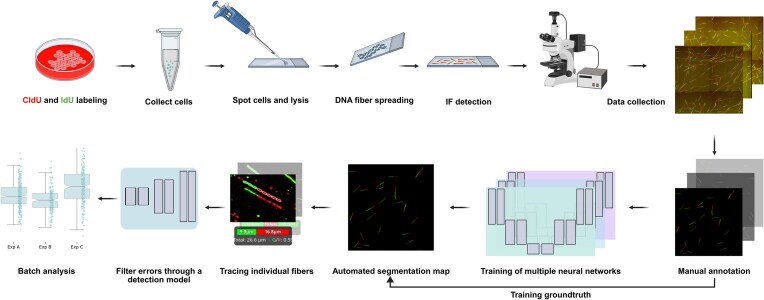

## Introduction

During the S phase of the cell cycle, cells undertake the critical task of faithfully replicating their genetic material. DNA replication is initiated at chromosomal sites called origins, from which two replication forks proceed in opposite directions along the template DNA (reviewed in [1–3]). DNA replication fork progression can be impeded by a plethora of environmental or endogenous genotoxic agents, resulting in a state of replicative stress [4–8]. Interestingly, several developmental syndromes, e.g. primordial dwarfism, have been shown to be caused by mutation in DNA replication factor-encoding genes and in mediators of the replicative stress response [9–15]. Moreover, replicative stress is a well-known driver of cancer-associated mutagenesis and response to genotoxic chemotherapy drugs [4,16,17]. Consequently, the characterization of DNA replication dynamics is the focus of intense research interest.

Experimental studies of replication have relied extensively on labeling nascent DNA strands with halogenated deoxyuridine analogs, including chloro-deoxyuridine (CldU), bromo-deoxyuridine (BrdU), and iodo-deoxyuridine (IdU). Cells are incubated with media containing these exogenous nucleoside analogs that are rapidly incorporated into replicating DNA. The dynamics of incorporation of these analogs can be analyzed using immunofluorescence (IF) strategies combined with flow cytometry or microscopy. Among these methods, DNA fiber spreading and combing [18–20] rely on the stretching of DNA molecules on glass slides or coverslips, followed by IF microscopy to visualize and measure individual analog-labeled tracks in replicated DNA. Sequential incorporation of the analogs enables identification and quantification of progressing replication forks, replication origins, and termination sites. Measurement of individual DNA fiber length provides information on the dynamics and rate of replication fork progression. Because of these capabilities, DNA fiber analysis has established itself as an indispensable tool for studying DNA replication.

DNA fiber analyses have been hampered by the tedious and labor-intensive process of manually measuring the length of labeled fibers from IF images. While a handful of studies have attempted to automate this task using standard image analysis software pipelines, none of these solutions gained widespread adoption. This is likely due to their sensitivity to the quality of DNA fibers IF images, leading to a high rate of errors, biases, and overall limited reliability. We previously proposed a DNA fiber measurement pipeline relying on classical computer vision techniques [21]. It involved identifying sparsely populated regions through DNA fiber density estimation, segmentation using a modified Canny edge detector, and diverse post-processing steps such as termination point reconnection and small blob removal. While conceptually sound, this approach tends to erroneously detect and segment too many fibers, especially in noisy or densely populated images. ‘DNA Stranding’, introduced by Li *et al.* [22], follows a similar approach and wraps the algorithm in a semi-automatic software providing a user-friendly interface. Users can refine segmentation results, offering a balance between automation and manual selection and measurement of fibers.

In the past decade, deep learning has established new standards in terms of segmentation performances [23]. While the use of these approaches for biomedical image analysis has gained considerable traction due to their improved generalization capabilities, no software solutions specifically adapted for DNA fiber analysis have yet been published in peer-reviewed journals. Here, we present an experimentally validated, free, open-source, and user-friendly tool that allows the segmentation, enumeration, and measurement of labeled DNA fibers from IF microscopy images obtained using the DNA spreading technique with two sequential pulses of nucleoside analogs. Our pipeline, called DNAi, integrates deep-learning-based segmentation, various post-processing steps, and visualization in a unified framework. The system is designed for usability and transparency, and provides a graphical interface that enables users to configure parameters and validate results interactively.

## Materials and methods

### Cell culture

U2OS and HeLa cells (purchased from ATCC), were cultured in Dulbecco’s modified Eagle medium (Gibco/Thermo Fisher, CAT #11995065) supplemented with 10% fetal bovine serum (FBS; Wisent, CAT #098150), $5 \,\mathrm{m}\mathrm{M}$ L-glutamine (Gibco/Thermo Fisher, CAT #25030081), and antibiotics ($100 \,\mathrm{\mu}\mathrm{g}\mathrm{m}^{-1}\mathrm{l}$ penicillin and $100 \,\mathrm{\mu }\mathrm{g}\mathrm{m}^{-1}\mathrm{l}$ streptomycin; Gibco/Thermo Fisher, CAT#15140122). PEO1 and PEO4 cell lines were shared by the laboratory of Alexandre Maré chal (Université de Sherbrooke). These cells were cultured in ovarian surface epithelium medium (OSE; Wisent, CAT #316-030-CL) supplemented with 10% FBS (Wisent), $5 \,\mathrm{m}\mathrm{M}$ L-glutamine (Gibco/Thermo Fisher), and antibiotics ($100 \,\mathrm{\mu }\mathrm{g}\mathrm{m}^{-1}\mathrm{l}$ penicillin and $100 \,\mathrm{\mu }\mathrm{g}\mathrm{m}^{-1}\mathrm{l}$ streptomycin; Gibco/Thermo Fisher). Cells were cultured at $37 \,\mathrm{^{\circ }C}$ in 5% $\mathrm{CO}_2$. Cell lines were tested for the absence of mycoplasma contamination using the ZmTech Mycoplasma polymerase chain reaction detection kit (#M209001).

### Small interfering RNA (siRNA) transfection

Cells were transfected with siRNA (Table 1) using Lipofectamine RNAiMax (Thermo Fisher, CAT #13778500) following the manufacturer’s instructions. The culture medium was changed after 24 h, and DNA fiber assays were performed 48 h after transfection.

**Table 1.** List of siRNA used in this study

Name	Supplier	Catalog	Sequence
siRNA siNT	Sigma–Aldrich	Custom	UGGUUUACAUGUCGACUAA [dT][dT]
			UGGUUUACAUGUUGUGUGA [dT][dT]
			UGGUUUACAUGUUUUCUGA [dT][dT]
			UGGUUUACAUGUUUUCCUA [dT][dT]
siBRCA1#1	Sigma–Aldrich	SASI_Hs01_00179500	CUACUGUCCUGGCUACUAA
siBRCA1#2	Sigma–Aldrich	SASI_Hs02_00343364	CAUUUAAACGCCACCAAUU
siBRCA1#3	Sigma–Aldrich	SASI_Hs01_00179502	CUAUGCAAGGGUCCCUUAA
siBRCA1#4	Sigma–Aldrich	SASI_Hs01_00179501	GACAAUGGCUUCCAUGCAA
siBRCA2	Dharmacon SMART pool	L-003462-00-0005	
si53BP1	Dharmacon	D-003548-04-0002	GAUAUCAGCUUAGACAAUU

### DNA fiber spreading

DNA fiber spreading assays were performed as previously described with slight modifications [24]. Briefly, cells were seeded at a density $5 \times 10^5$ cells per 6-cm dish and cultured overnight at $37 \,\mathrm{^{\circ }C}$ in 5% $\mathrm{CO}_2$. Cells were pulse-labeled with $30 \,\mathrm{\mu }\mathrm{M}$ 5-chloro-2’-deoxyuridine (CldU, Sigma–Aldrich, CAT #C6891), washed twice with phosphate buffered saline (PBS), and then incubated with $250 \,\mathrm{\mu }\mathrm{M}$ 5-iodo-2’-deoxyuridine (IdU, Sigma–Aldrich, CAT #I7125) at $37 \,\mathrm{^{\circ }C}$. The time and order of incubation are indicated in the corresponding figure legends.

After nucleoside analog pulses and drug treatment, cells were harvested by trypsinization, washed once with ice-cold PBS, and resuspended in PBS. Cells were spotted onto microscope slides, lysed with DNA lysis buffer ($50 \,\mathrm{m}\mathrm{M}$ ethylenediaminetetraacetic acid, 0.5% sodium dodecyl sulphate in $200 \,\mathrm{m}\mathrm{M}$ Tris–HCI, pH = 7.5), and tilted to stretch DNA fibers. Fibers were fixed in freshly prepared cold methanol/acetic acid (3:1, v/v) for $10 \,\mathrm{min}$, air-dried, and either stored at $4 \,\mathrm{^{\circ }C}$ or processed immediately for IF. Slides were treated with $2.5 \,\mathrm{M}$ HCl for $80 \,\mathrm{min}$, rinsed three times in PBS, and blocked with 5% bovine serum albumin (BSA) in PBS for $20 \,\mathrm{min}$ at $37 \,\mathrm{^{\circ }C}$. Incorporated nucleotide analogs were detected using rat anti-BrdU antibody recognizing CldU (Abcam, CAT #ab6326; 1:400) and mouse anti-BrdU antibody recognizing IdU (BD Biosciences, CAT#347580; 1:25). Secondary antibodies were Alexa Fluor 594-conjugated goat anti-rat IgG (1:100; Thermo Fisher, CAT #A11007) and Alexa Fluor 488-conjugated goat anti-mouse IgG (1:100; Thermo Fisher, CAT #A11029). All antibody incubations were performed in a humid chamber at room temperature, followed by three washes in PBS-T (PBS with 0.05% Tween-20) and one wash with PBS. Slides were mounted with Immuno-Fluore mounting medium (MP Biomedicals, CAT#622701) and stored at $4 \,\mathrm{^{\circ }C}$ or $-20 \,\mathrm{^{\circ }C}$ in the dark until imaging.

### Genotoxic treatment

The following treatments were used in this study: hydroxyurea (HU; Bio Basic Canada Inc. CAT#HB0528), ATR inhibitor [ATRi; VE-821, ApexBio (Cedarlane), CAT #A2521-5.1], olaparib [Cayman Chemical (Cedarlane), CAT #10621-25]. Olaparib and the ATR inhibitor were dissolved in Dimethyl sulfoxide (DMSO, Sigma-Aldrich, #D8418), which was used as a control in corresponding experiments. Treatment conditions are indicated in the corresponding figures. For ultraviolet (UV)-induced replication stress, cells were exposed to UV ($25 \,\mathrm{J}\mathrm{m}^{-2}$) by irradiation with monochromatic $254 \,\mathrm{n}\mathrm{m}$ UV using a G25T8 germicidal lamp (Philips). The fluence was measured with a Spectroline DRC 100$\times$ digital radiometer equipped with a DIX-254 sensor (Spectronics Corporation).

### S1 nuclease assay

DNA fiber assays with S1 nuclease treatment were performed as described in the DNA fiber spreading section, with minor modifications. Cells were labeled with $100 \,\mathrm{\mu }\mathrm{M}$ CldU (Sigma–Aldrich, CAT #C6891) for the duration indicated in the corresponding figure legend, followed by labeling with $250 \,\mathrm{\mu }\mathrm{M}$ IdU for the specified time.

Following labeling, cells were permeabilized with CSK100 buffer ($100 \,\mathrm{m}\mathrm{M}$ NaCl, $10 \,\mathrm{m}\mathrm{M}$ MOPS pH 7.2, $3 \,\mathrm{m}\mathrm{M}$ MgCl$_2$, $300 \,\mathrm{m}\mathrm{M}$ sucrose, 0.5% Triton X-100) for $10 \,\mathrm{min}$ at room temperature. Cells were then treated with S1 nuclease (Thermo Fisher, CAT #EN0321) at $20 \,{\rm U}/{\rm ml}$ in S1 buffer ($30 \,\mathrm{m}\mathrm{M}$ sodium acetate, pH 4.6, $10 \,\mathrm{m}\mathrm{M}$ zinc acetate, $50 \,\mathrm{m}\mathrm{M}$ NaCl, 5% glycerol) for $30 \,\mathrm{min}$ at $37 \,\mathrm{^{\circ }C}$. Control samples were processed in parallel without S1 nuclease.

Cells were collected by gentle scraping in PBS containing 0.1% BSA, and nuclei were pelleted by centrifugation at $4600$ $\times \, g$ for $10 \,\mathrm{min}$ at $4 \,\mathrm{^{\circ }C}$. After removal of the supernatant, nuclei were resuspended to a final concentration of ∼$2000 \,{\rm nuclei } / \mathrm{\mu }\mathrm{l}$ and processed for DNA spreading as described previously.

### Microscopy platforms

Images were collected using a 63$\times$/1.4 objective lens on a widefield fluorescence Zeiss Axio Imager Z2 microscope with Zeiss Axiocam 820 monochrome sCMOS camera, a DeltaVision Elite System (GE Healthcare) using an Olympus 60$\times$/1.42 objective and a Leica Stellaris confocal microscope (Leica Microsystems) equipped with an HC PL APO CS2 63$\times$/1.40 objective and HyD detectors. Zeiss Zen blue software was used to capture Zeiss images, Resolve3D softWoRx-Acquire for DeltaVision and LAS X software for Leica images.

### Training data annotation

Training data were obtained by manually labeling ongoing forks and replication origins (at a pixel level) on tiles extracted from whole-slide images acquired with a Zeiss Z2 Axio Imager microscope. In total, we sampled 1795 nonoverlapping tiles of size $2048\times 2048$ pixels. These were divided into a training set of 1536 tiles and a testing set of 259 tiles, with 20% of the training set reserved as a validation set.

**Figure 1.**
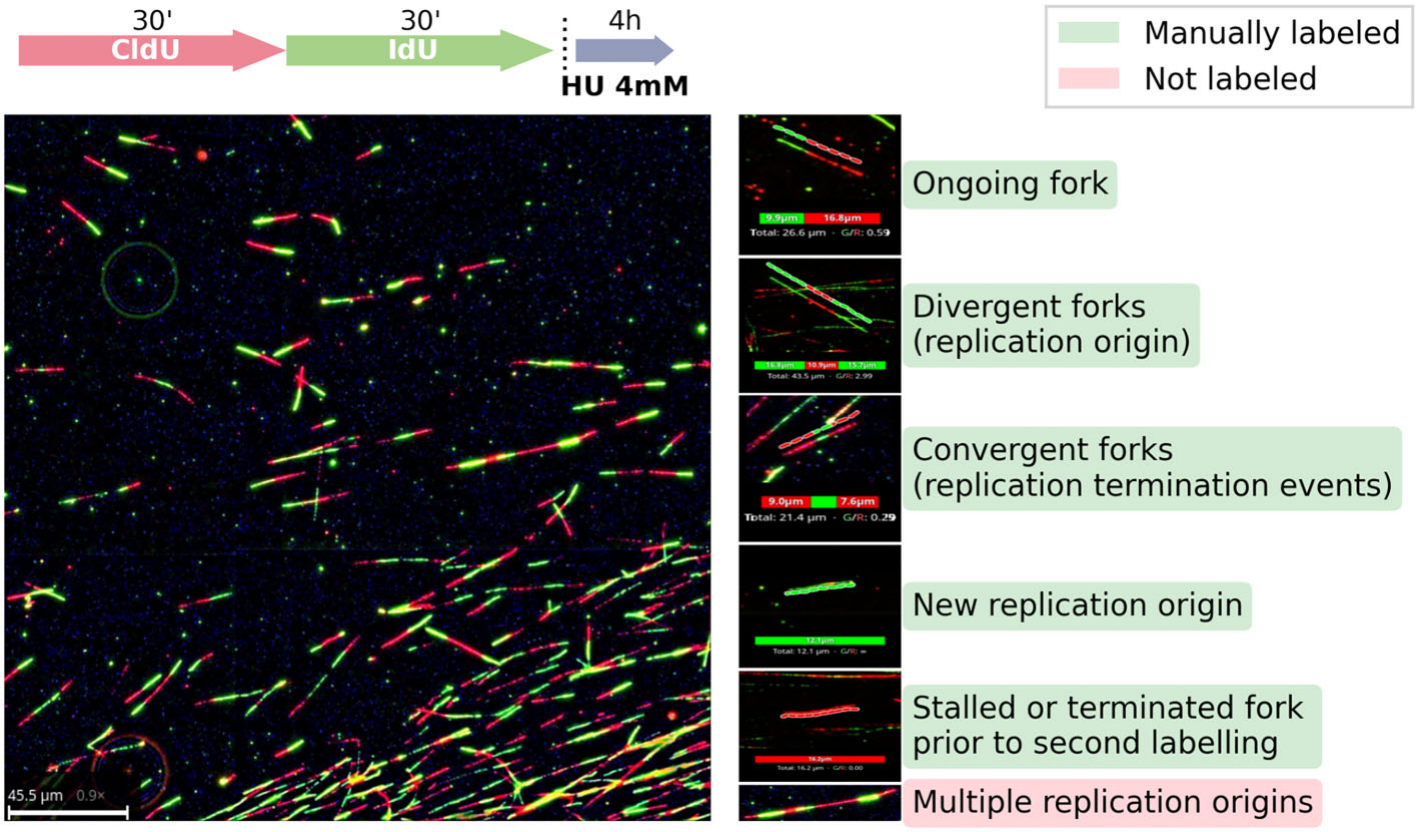
Example of manual DNA fiber labeling performed to generate the training and validation datasets. Top schema: labeling protocol used in this particular experiment. In cases of ambiguity in the analog signal (e.g. mixed colors or yellowish appearance at the transition between colors), annotators were instructed to assign the segment to the second analog (IdU).

Four annotators participated in the annotation campaign, manually tracing all bicolor fibers they identified in each tile. To assess inter-annotator consistency, 20 tiles were independently labeled by all four experts. All annotations were performed using an in-house, open-source software called *LabelMed* (github.com/ClementPla/Annotator/). Annotators were instructed to trace fibers as accurately as possible using a pencil tool of fixed radius (3 pixels). In cases of ambiguity during color transitions (e.g. fibers appearing yellow), annotators were instructed to assign the pixel to the second label (i.e. green). A representative example of an annotated image is represented in Fig. 1. In total, 14 728 fibers were labeled (11 495 double, 1893 triple, and 1340 single segments).

### DNAi pipeline

#### Preprocessing

To ensure uniformity and compatibility with our deep learning pipeline, several preprocessing steps were applied to all images. The spatial resolution was standardized by rescaling each image to a fixed scale of 0.26 μm per pixel. While this scale was fixed during training, the pixel size is configurable in the final tool to accommodate images from different acquisition systems. The preprocessing pipeline normalizes raw fluorescence images to correct for uneven illumination and to enhance fiber contrast. Given an input image, the algorithm proceeds as follows:

1. **Background modeling**. The image is normalized to [0,1] per channel. A median filter is then applied to a downsampled proxy to estimate the local background, thereby avoiding full-resolution floating-point computation.
2. **Background subtraction**. The background is subtracted with a user-controlled clarity factor $\alpha \in [0, 1]$ that modulates subtraction strength. All experiments presented here are conducted with the default clarity factor of 1.0.
3. **Contrast rescaling**. The corrected intensities are rescaled so that the $p_{99\alpha }$ percentile maps to the maximum display value, estimated via strided sampling to limit memory usage. The result is clipped and cast to 8-bit unsigned integers.

To further increase the robustness of the model and reduce overfitting, we applied a series of data augmentation techniques during training. These included random image rotations, horizontal and vertical flips, and rescaling, which collectively helped diversify the training examples without altering the underlying structures.

#### Segmentation model

To identify the most effective approach for DNA fiber segmentation, we evaluated several neural network architectures, ranging from well-established convolutional models to state-of-the-art transformer-based designs. Classical architectures such as the CNN-based U-Net [25] were included due to their proven performance in biomedical image segmentation tasks, with different encoders [26–28]. We also explored more recent architectures like SegFormer [29], which leverages a transformer-based encoder to better capture long-range dependencies and contextual information across the image. We used transfer learning, as each of the segmentation models had its encoder pretrained on ImageNet [30]. As a loss function, we used the Generalized Dice Loss, which was found to offer good performance, likely because of the very imbalanced class distributions. Each model was trained for 1000 epochs, with the AdamW optimizer, a learning rate automatically tuned before each training on 100 iterations, and a weight decay of 0.0001.

#### Post-processing

Following segmentation, post-processing steps were applied to refine the predicted fiber structures and enable accurate downstream analysis.

##### Junction disentanglement

The first step involved skeletonization, where the segmented fibers were reduced to their 1-pixel-wide centerlines, using an endpoint-preserving skeletonization algorithm applied to each connected component individually. This process is required to identify junctions (points where fibers intersect or branch) using a hit-and-miss transform that tests each pixel neighborhood against predefined intersection templates. Nearby junction pixels were then merged via single-linkage hierarchical clustering (threshold of 10 pixels) to consolidate complex intersection regions into single junction locations. Each clustered junction was masked out by erasing a circular region proportional to the estimated fiber width, splitting the skeleton into isolated branches. Branches shorter than a minimum length threshold were discarded.

**Figure 2.**
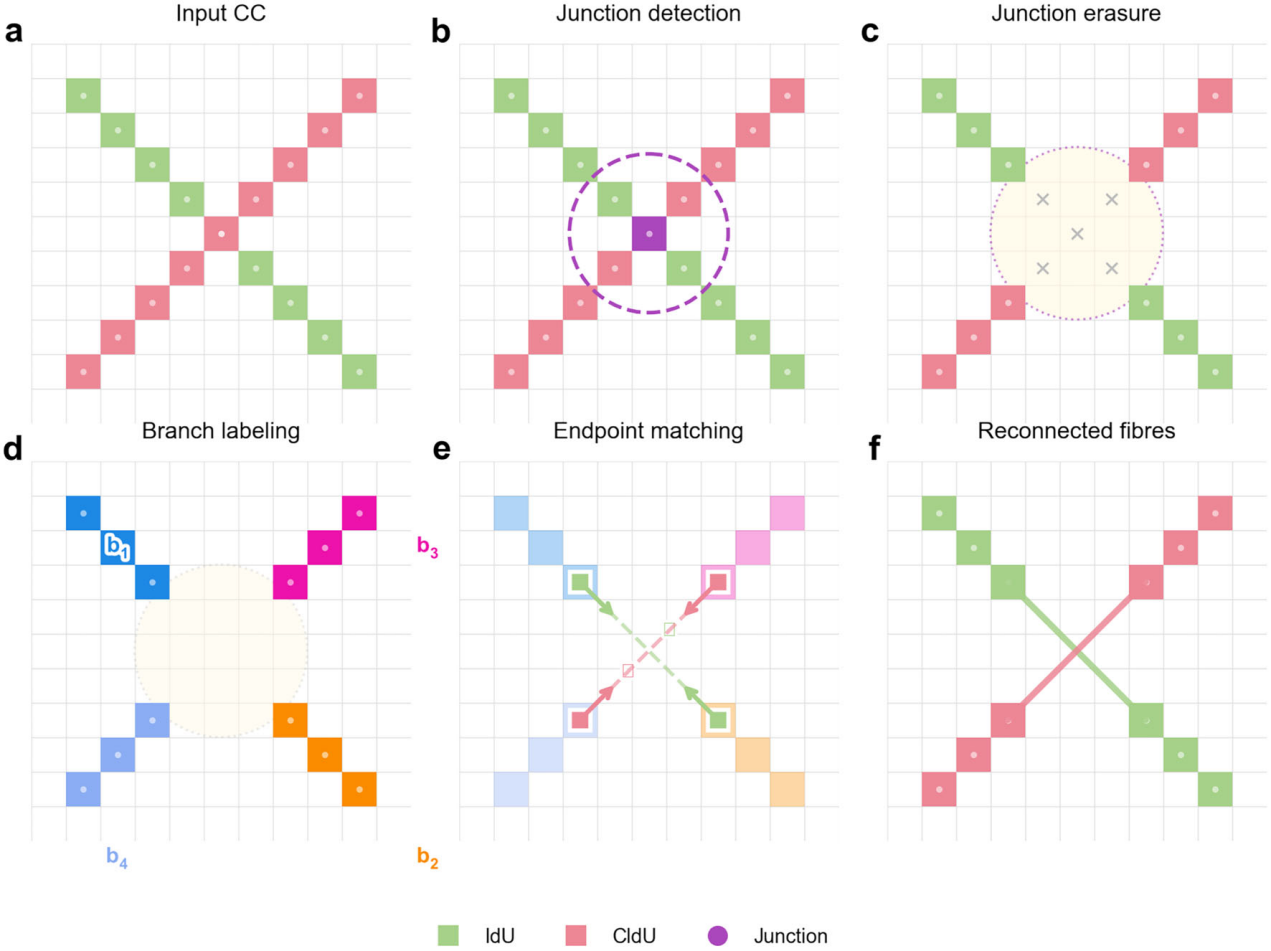
Post-processing steps taken to reconcile junctions in the segmentation. Junctions identification and removal **(A, B)**, junction erasure in a fixed radius **(C)**, branch orientation estimation **(D)**, endpoint matching and fiber reconnection **(F)**.

To determine how branches should be reconnected across junctions, endpoint detection was performed on the remaining skeleton, and each endpoint’s directional angle was estimated by tracing 15 pixels along the skeleton path. Endpoints were then matched at each junction using a weighted cost function combining three criteria: spatial proximity (normalized by a maximum distance proportional to fiber width), angular compatibility (favoring endpoints whose directions are ∼180$^{\circ }$ apart, indicating collinear continuation), and color consistency (penalizing connections between segments of different labels). The matching strategy adapts to junction topology: two-endpoint junctions are directly paired, three-endpoint junctions (T-junctions) select the best single pair while the unmatched endpoint is connected to the junction center, and four-endpoint junctions (crossings) evaluate all three possible pairings to find the globally optimal assignment. Reconnection lines are drawn between matched endpoints, with a green label assigned when the two connected branches carry different analog labels; otherwise, the shared label is preserved. Unmatched T-junction tips are reconnected to the junction center using their own label (Fig. 2).

Finally, the reconnected branches are grouped into fibers by building a branch adjacency graph from the established pairings and extracting its connected components. To eliminate noise introduced during segmentation and junction processing, reconstructed fibers that consist of a single label or whose total length falls below 15 pixels are discarded.

##### Error filtering

We implemented an optional filtering step to automatically remove aberrant segmentation towards improving the quality of the resulting quantifications with certain images. This solution relied on a secondary neural network that processed each candidate fiber individually. For every detected fiber, we extracted its bounding box and stacked the raw image patch with its corresponding segmentation mask as input channels. In addition, we feed the model with a vector of features extracted from the segmented fiber: its tortuosity and curvature, which help identify anomalously curved or nonlinear structures unlikely to represent true fibers, and the mean intensity of the red and green channels, which help reject artifacts such as holes or faintly stained regions that lack the expected fluorescence signal. The classifier is trained as a binary decision model to distinguish between valid fibers and false positives. Given the relative simplicity of this binary classification task, the secondary network provided an efficient mechanism to automatically refine the segmentation output, ensuring that only high-quality fibers were passed to subsequent analysis stages. We trained the error detection model on 2,968 fibers, of which 21.9% corresponded to manually identified errors. Following hyperparameter optimization, a pretrained Convnext-tiny architecture was selected as a suitable backbone for false-positive detection.

##### Final cleaning

In the final step, we discarded all fibers that are not bicolor, that contained >3 segments, as well as those located at the image borders to avoid including cropped fibers.

### Inference

To efficiently process large stitched mosaics of whole slides, we implemented a sliding window approach to enable full-image segmentation. In this strategy, each large mosaic is divided into smaller overlapping patches of 1024 $\times$ 1024 pixels, with a 10% overlap between adjacent patches. This overlap ensures continuity and consistency at the boundaries, reducing edge artifacts and improving the quality of the assembled segmentation. Following model inference on each patch, the individual predictions are reassembled into a full-resolution mask. To minimize the influence of border predictions, a Gaussian weighting function is applied during reconstruction. This approach assigns greater importance to predictions near the center of each patch, where the model’s output is typically more reliable, while softly blending overlapping regions to ensure smooth transitions across the image. Once this initial segmentation is obtained, the post-processing is done on the CPU. We used Numba to accelerate our code by precompiling Python into optimized machine code.

Finally, we implemented two additional options within DNAi: ensembling and test-time augmentation (TTA) [31]. Both techniques produce multiple outputs (which, in the case of a segmentation task, are per-pixel probabilities), which are then recombined into a single prediction by pixel-wise averaging. The former consists of several independently trained models whose segmentation maps are averaged, thereby reducing variance and improving robustness compared to a single model. TTA addresses a fundamental challenge in deep learning: most architectures are not inherently equivariant to geometric transformations, meaning that changes in image orientation or scale can affect segmentation performance. While training-time data augmentation helps the model learn diverse representations, it cannot formally guarantee equivariance. TTA mitigates this limitation at inference time by applying a predefined set of geometric transformations (horizontal flips, vertical flips, and 90$^{\circ }$ rotations) to each test image, obtaining predictions for each augmented version, transforming them back to the original image space, and aggregating them by averaging. This ensemble over geometric configurations helps average out orientation-dependent biases, producing more robust and consistent segmentations without requiring retraining, at the cost of increased inference time proportional to the number of applied transformations.

### Algorithmic efficiency

**Figure 3.**
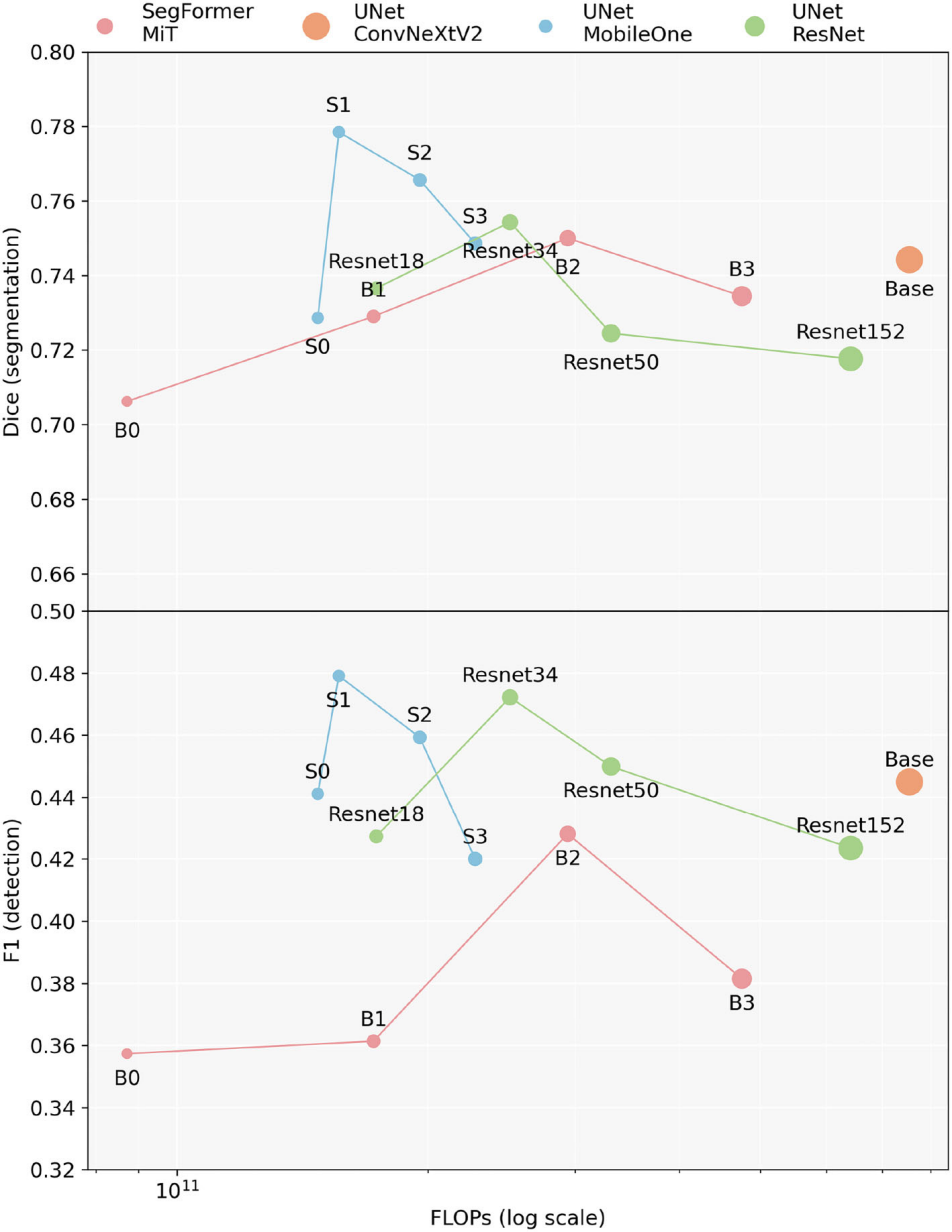
Comparison of several model variants (names indicated above each points of the graph), organized from smaller to larger architectures. The top panel shows segmentation performance, measured as the multiclass Dice score, plotted against the FLOPs (floating-point operations required to process an image of size 1024 $\times$ 1024). The bottom panel illustrates detection performance, measured by the F1 score, defined as the harmonic mean of detection precision and recall. The size of each variant (number of parameters) is represented by the respective radius of each point on the graph.

The efficiency of different model families chosen among recent state-of-the-art was achieved by comparing the evolution of segmentation (Dice) and detection performance as a function of the number of floating-point operations (FLOPs) required for the forward pass of a $1024 \times 1024$ image. Detection metrics quantify how well DNAi identifies individual fibers compared to the human annotator. Detection precision is computed as the number of correctly matched fibers divided by the total number of fibers predicted by DNAi, while detection recall is the number of correctly matched fibers divided by the total number of ground-truth fibers. The detection F1 score, defined as the harmonic mean of precision and recall, provides a single balanced measure of detection performance. A predicted fiber is considered correctly matched to a ground-truth fiber if their intersection-over-union exceeds a threshold of 0.5, with matches assigned greedily by decreasing IoU to enforce one-to-one correspondence. The size of each variant (number of parameters) is represented by the respective radius of each point on the graph (Fig. 3). All model variants are made available to the end-user, allowing a trade-off between computational cost and performance, adaptable to different hardware constraints.

## Results

### Overview

We present a novel system called DNAi for the automated analysis of DNA fiber assays, leveraging deep learning for the segmentation of IF images. At the core of the pipeline is a neural network trained to identify pixels in IF microscopy images that correspond to DNA fibers containing consecutive, adjacent segments marked by nucleoside analogs (i.e. bicolor fibers). To enhance model robustness and generalizability, the network was trained on a diverse collection of IF images spanning a broad range of experimental conditions. These included experiments designed to assess nascent DNA stability, including replication fork protection and S1 nuclease assays, as well as measuring replication fork progression under replication stress induced by HU, olaparib, or UV treatment. Human cells of various origins were sequentially labeled with CldU and IdU, with IdU incorporation times ranging from 30 to 90 min, in the presence or absence of genotoxic agents depending on the experimental design (see the ‘Materials and methods’ section for details). For S1 nuclease assays, cells were additionally treated with S1 nuclease following genotoxic exposure to reveal single-stranded DNA (ssDNA) gaps generated under replication stress. Images used for training and validation experiments presented below were acquired using a Zeiss Z2 Imager system, unless otherwise indicated (see the ‘Materials and methods’ section for details).

In preparing our training dataset, we annotated DNA fibers by measuring individual CldU or IdU tract lengths, as well as adjacent CldU–IdU segments, including single color, bicolor ongoing forks and convergent/divergent fork structures (Fig. 1). The total length (CldU+IdU) of annotated DNA fiber segments ranged from $3.51 \,\mathrm{\mu }\mathrm{m}$ to $88.58 \,\mathrm{\mu }\mathrm{m}$, with individual segment length varying from $0.36 \,\mathrm{\mu }\mathrm{m}$ to $57.32 \,\mathrm{\mu }\mathrm{m}$ and ratio values from 0.25 to 36.1 with a median of 1.20. We used this spectrum of DNA fiber characteristics with the goal of generating a network capable of segmenting and measuring a wide variety of segment lengths and ratios of labeled segments, irrespective of the specific labeling conditions used by the experimenter. We focused our efforts on ensuring robust detection, segmentation, and quantitative measurement of DNA fibers containing two or three adjacent labels of distinct colors in the final image (Ongoing forks, Divergent forks, and Convergent forks in Fig. 1). As a consequence, detection and measurement of single-color fibers were not optimized and validated, and their assessment has not been made available to the end-user.

We evaluated several neural network architectures, comparing their efficiency and performance for this task. As described in detail in the ‘Materials and methods’ section, our pipeline includes post-segmentation tools for disentangling overlapping fibers and tracing individual ones to determine their length, identifying the fiber type, and IdU/CldU ratio. To further align automated outputs with human interpretation, we included an optional filtering module trained to detect false positives from the initial segmentation. The entire workflow is accessible through a user-friendly interface that supports configuration of key steps and interactive visualization of results.

Unless otherwise specified, all reported results are obtained using the ensemble models with TTA, followed by the error detection module to filter out unacceptable fibers. The impact of these two components is examined in more detail in the following section.

### Inter-annotator variability and algorithm performance

**Figure 4.**
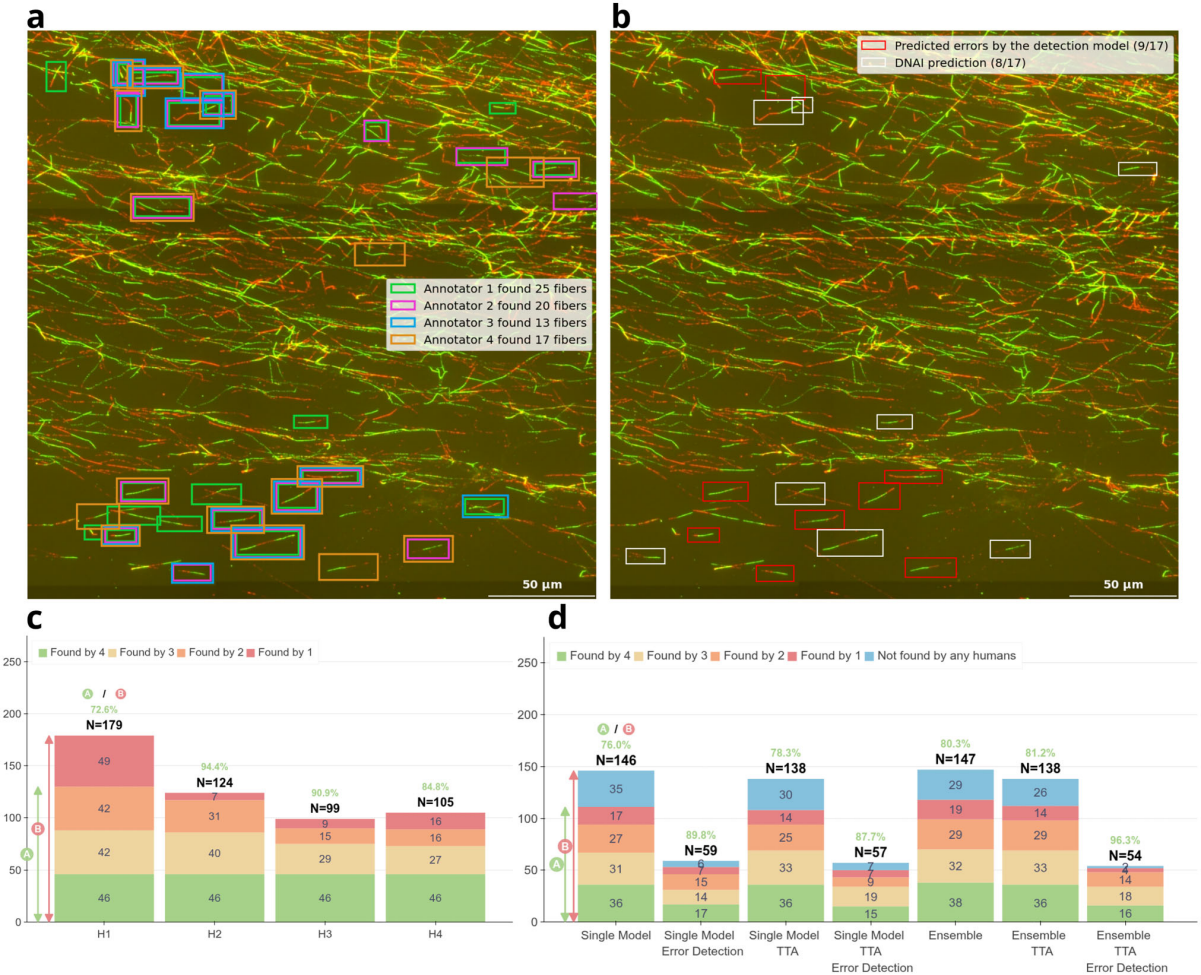
Inter-annotator variability. (**A**) Example of an image labeled by four annotators. (**B**) Segmentation of DNA fibers using DNAi with or without the error detection model. (**C**) For each human annotator $H_i$, the plot shows the number of fibers that $H_i$ found in common with zero, one, two or three of the others annotators. The count is obtained on the subset of the test set composed of 20 images labeled by the four annotators. (**D**) Comparison of the fibers found by different variants of DNAi and the humans annotators, with or without TTA, ensemble, and error detection model. The single model used is UNet-MobileOne S1.

DNA fiber IF images display highly variable quality and complexity due to factors related to both the acquisition itself (confocal or camera-based, sensor type, bit-depth, etc.) and the arrangement of DNA fibers (spatial density, alignment, overlap, etc.). On a set of 20 images independently annotated by four experts, we measured the number of fibers found by each. Figure 4**A** and **B** provides an example of the type of variability found in manual annotations and in the prediction made by the algorithm, with or without filtering. We counted, for each annotator, the occurrence of fibers present in 1, 2, or 3 of the other annotations. The histogram in Fig. 4C illustrates the inter-observer variability in the task of fiber detection. A fiber is considered common to two annotators if a sufficient fraction of their skeleton pixels lie within a distance of 5 pixels of the other’s skeleton. The data indicate that out of a total of 225 distinct fibers detected by human annotators, only 46 were detected by all four observers (i.e. 20.4%), and on average each annotator identified 14.3% of fibers that no other annotator found. Measured against this union, individual annotator recall ranged from 44.0% to 79.6%, with an average of 56.3%, illustrating the substantial variability in fiber detection even among trained human graders. This is consistent with our previously published results indicating that DNA fiber image interpretation typically leads to significant inter-annotator bias [21].

The number of fibers detected by different variants of our models is presented in Fig. 4. UNet-MobileOne S1 (single model in Fig. 4D), the model which we found had the highest precision on detecting fibers, segments a large number of fibers, of which a majority (76.0%) are detected by at least one human. This frequency increases with TTA (78.3%), but in turns TTA slightly decreases the number of fibers detected. In this configuration, the precision improves significantly by adding the error-detection model (87.7%%). Ensembling models also improves the precision (80.3% alone, 81.2% with TTA, and 96.3% with error detection), as represented in Fig. 4D.

**Figure 5.**
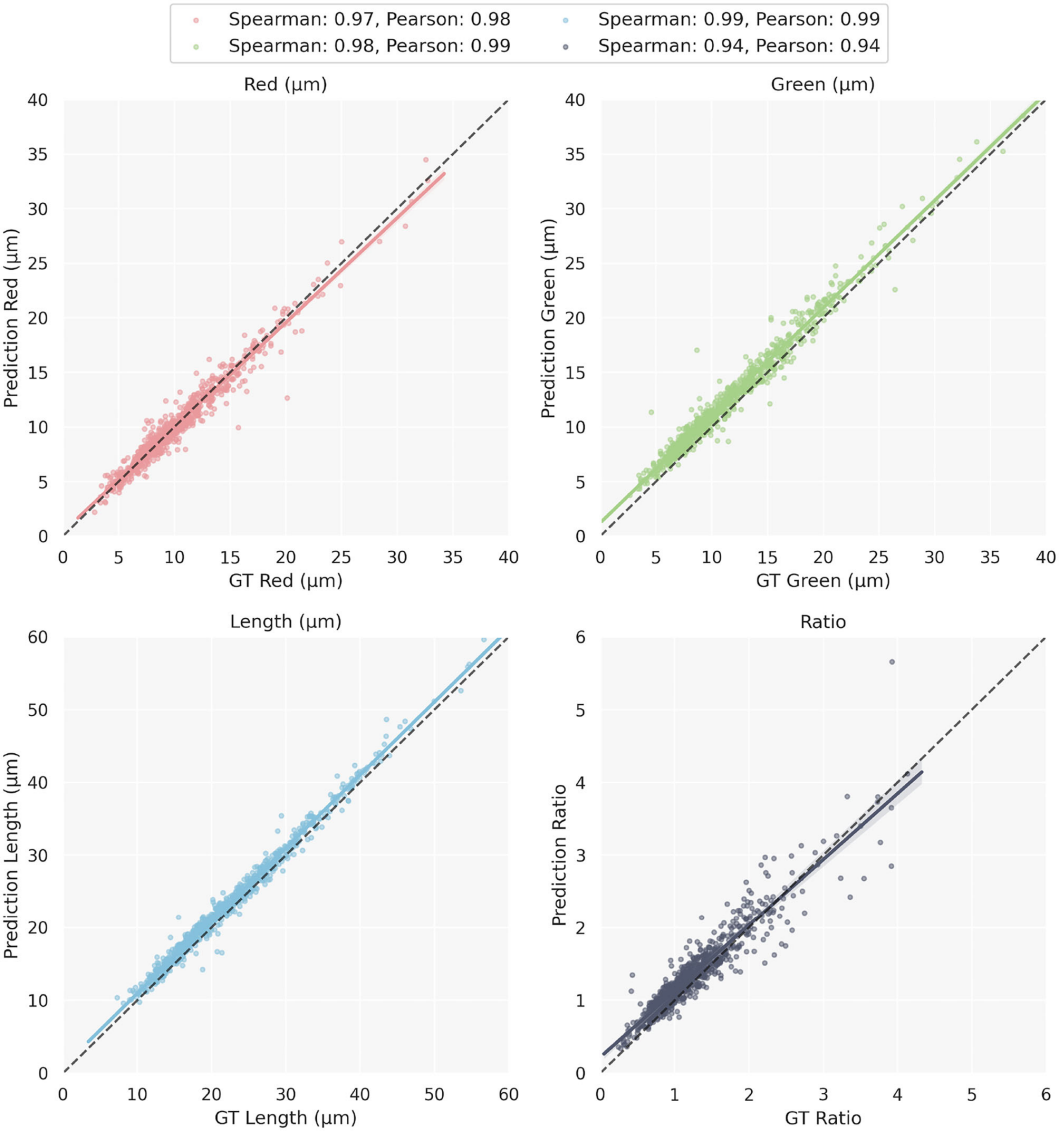
Comparison of fiber properties in the groundtruth (GT, the human annotator) versus DNAi. The regression was computed only on fibers detected by both (*N* = 769).

To further validate the segmentation performance of the model, we used a test set consisting of a total of 259 images. This allowed us to quantify the ratio between the length of CldU and IdU labeled segments in bicolor fibers. Figure 5 shows the concordance of the ratios measured in the 769 fibers found by both human and DNAi. Among these fibers, we observe an excellent agreement between the human measurement and that of the algorithm, indicating that DNAi closely reproduces human segmentation. Thus, whenever humans and DNAi agreed on the detection of a fiber, their respective measurements (lengths and ratios) were similar.

**Figure 6.**
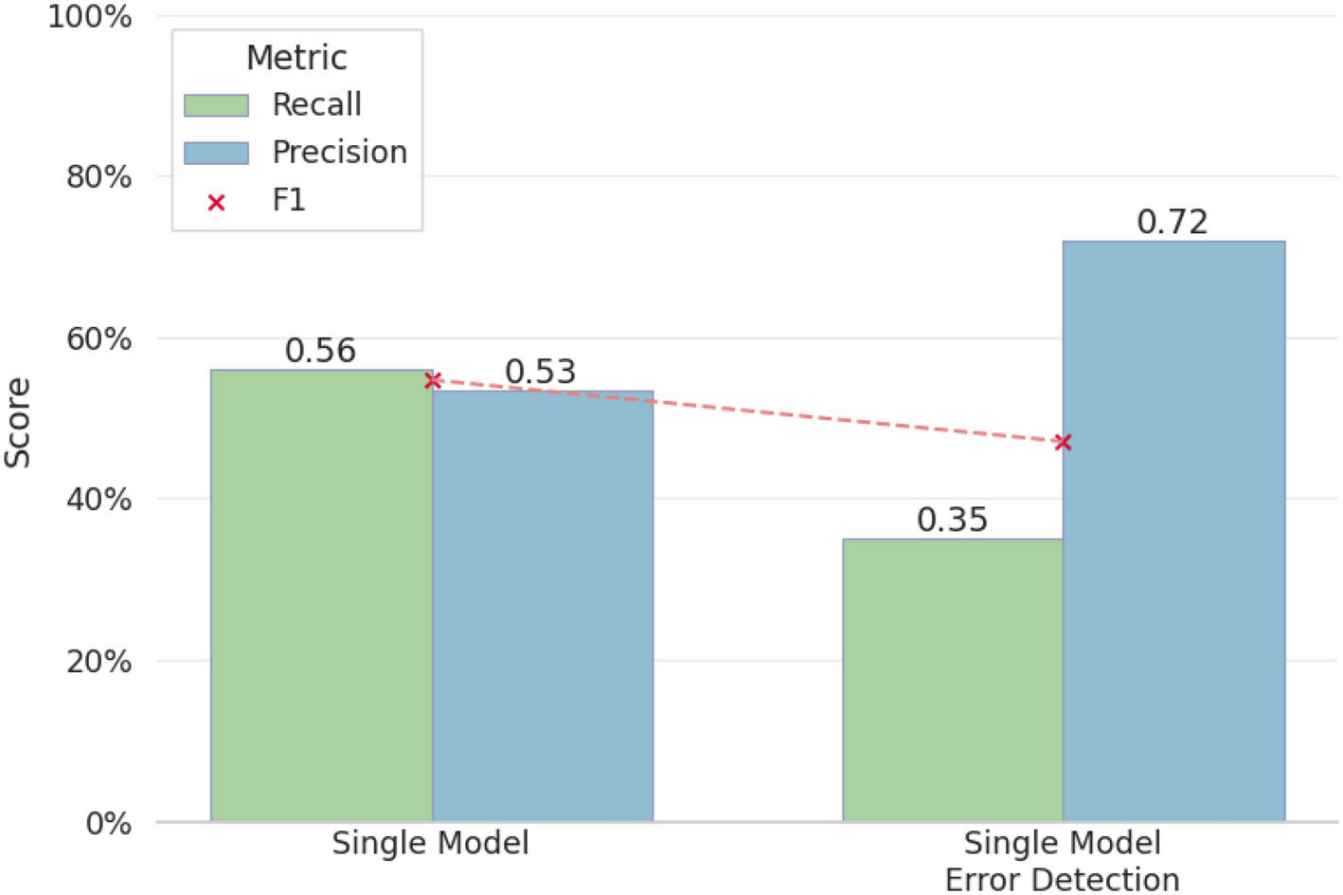
Detection performance (precision, recall, and F1) evaluated on 259 test images, using the UNet-MobileOne S1 model (Single model) with and without the error detection module. Enabling error detection substantially increases precision at the expense of recall, as candidate fibers with high predicted error probability are filtered out.

We next quantified how often such agreement occurs by evaluating precision and recall, comparing the same model with and without the error-filtering module. True Positives (TP) were defined as fibers in the predictions that also appeared in the ground truth, and False Positives (FP) as predicted fibers absent from the ground truth. We assess the error-filtering module on our full test set and report the results in Fig. 6: it improves precision at the expense of recall, as candidate fibers with high predicted error probability are filtered out. Similarly, TTA improves precision but reduces recall, as the averaging of multiple augmented predictions tends to suppress weakly detected fibers rather than introduce new ones. The choice of configuration is therefore situational: when maximizing fiber recovery is critical, the base model alone is preferable; when minimizing false positives is the priority, enabling error detection, TTA, or both offers a better trade-off, at the cost of increased computation for TTA, which requires multiple inference passes.

### Assessing the performance of DNAi on datasets with limited annotations

The previous results were obtained under controlled conditions typical of machine learning protocols, using datasets annotated consistently for both training and evaluation. The limited size of the test set prompted us to extend the evaluation to more diverse experimental scenarios. We validated DNAi on a larger set of images that were manually analyzed, and where only fiber-level ratio and label length measurements were available without any positional annotations on the images themselves. Images were processed and DNA fiber were manually measured using Image J (Fiji). We collected data from 57 biological condition, representing a total of 174 stitched images, conducted under various genetic silencing conditions leading to, according to manual analysis, median IdU/CldU ratios between 0.70 and 1.71, min/max ratio values of 0.11 and 5.68 and overall length ranging from $2.93 \,\mathrm{\mu }\mathrm{m}$ to $104.04 \,\mathrm{\mu }\mathrm{m}$.

**Figure 7.**
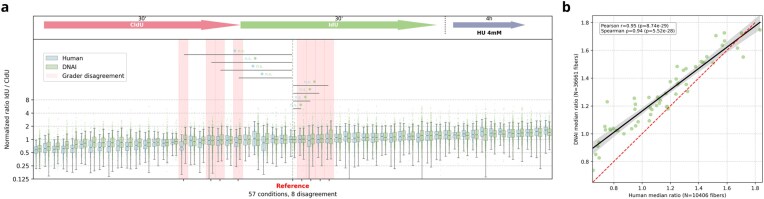
Evaluation of DNAi across 57 independent biological conditions, corresponding to a total of 174 stitched images. For each condition, a human annotator measured the ratio of around 200 fibers, providing a robust reference for comparison. Experiments were performed using either U2OS or HeLa human cells. **(A)** The labeling protocol used is illustrated schematically in the top panel. For each experiment and each grader (human versus DNAi), Cliff’s $\delta$ was computed against the reference condition with 95% bootstrapped confidence intervals. Disagreement (highlighted in red) is defined as one grader detecting a significant difference from the reference while the other does not. The median ratio value of the reference sample was also used to normalize values from all other samples, independently for each grader (human or DNAi). **(B)** Regression of the median ratios measured by DNAi against over those reported by a human annotator.

Each experiment included manual measurements of ∼200 fibers, resulting in a total of 10 406 manually measured fibers. In contrast, DNAi processing of the same dataset identified 36 661 fibers, as no upper limit was imposed on the number of fibers analyzed per experiment. The detected fibers spanned a wide range of lengths, from $1.17 \,\mathrm{\mu }\mathrm{m}$ to $102.96 \,\mathrm{\mu }\mathrm{m}$. Figure 7A illustrates the side-by-side comparison between human and DNAi for these 57 experiments. For each, we assessed whether each grader (human and DNAi) measured a median ratio that differed significantly from a reference condition (selected as the condition whose median ratio falls at the midpoint of all conditions). Concretely, for each experiment $i$ and each grader, we computed Cliff’s $\delta$ effect size between experiment $i$ and the reference, along with its 95% confidence interval obtained by bootstrapping. If the confidence interval contains zero, we concluded that the measured ratio was not significantly different from the reference, and vice versa. We then identified cases where the two graders reached divergent conclusions; one detecting a significant difference and the other not. Out of 57 experiments, such a disagreement occurred in only eight cases. Overall, the regression in Fig. 7B quantifies this excellent agreement, both in terms of linear correlation and relative ranking across conditions, with a Pearson’s correlation coefficient of 0.95 and a Spearman’s coefficient of 0.94. Nevertheless, we found that DNAi shows a systematic bias toward higher ratio estimates. We were unable to identify a single cause for this systematic overestimation. One contributing factor may be inter-grader disagreement on the precise location of the transition between the two analogs. Another potential source is the difference in length measurement methodology: human graders measure fiber length using polylines drawn between manually placed control points, whereas DNAi computes length along the skeletonized centerline, which may capture additional curvature and thus yield slightly longer measurements. Importantly, as shown in the figures below, even though the absolute ratio values are modestly increased in DNAi outputs compared to human graders, this increase occurs across all conditions within a given experiment, such that the relative differences between conditions remain similar to those obtained by manual measurements.

### Robustness to image degradation

To assess the robustness of the DNAi ensemble under suboptimal imaging conditions, we conducted a series of *in silico* experiments simulating two prevalent forms of image degradation. First, we applied Gaussian blurring with increasing standard deviation $\sigma$ to emulate out-of-focus acquisition. Second, we introduced additive Gaussian noise with progressively higher variance to simulate random pixel-level disturbances.

**Figure 8.**
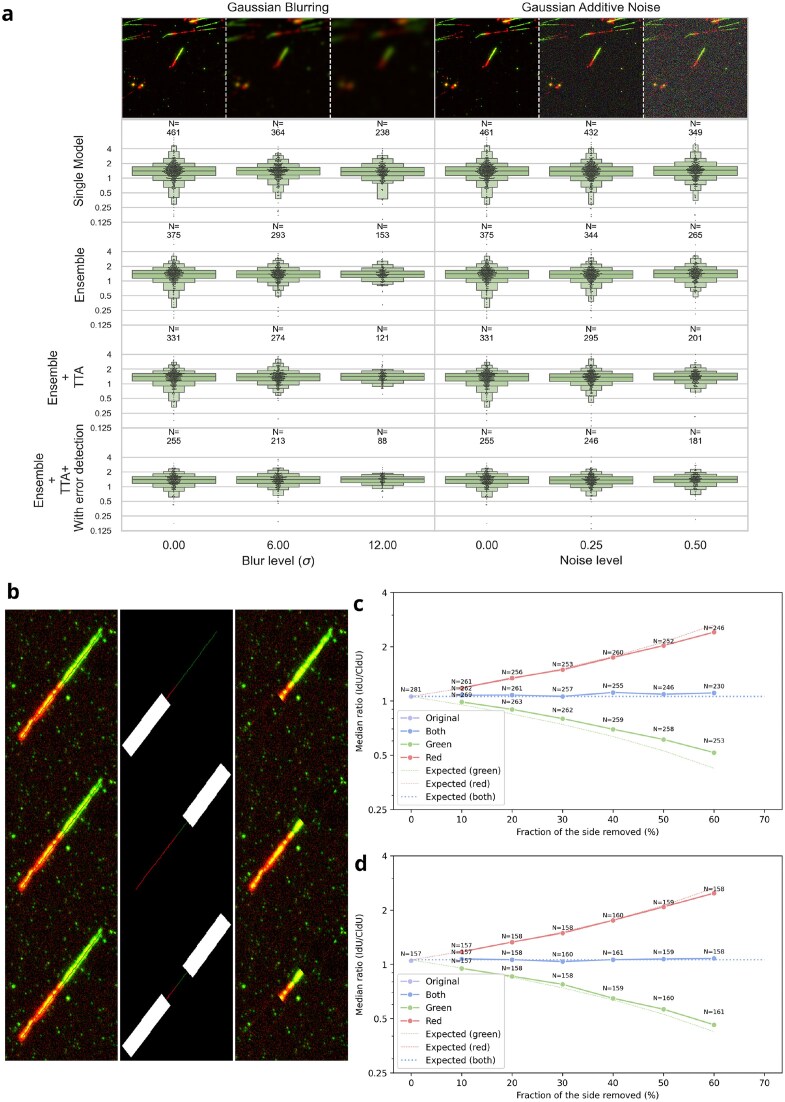
**(A)** Robustness of DNAi to image degradation. Images were progressively degraded using Gaussian blur (left) or additive noise (right). For each level of degradation, the median distribution of fibers detected by a single model, the ensemble, and the ensemble with TTA ± error detection is reported. (**B**) Schematic representation of the *in silico* strategy used to artificially shorten DNA fibers from one or both sides by a predefined fraction, with the cut regions reconstructed by inpainting (see text for details). (**C**) DNAi-measured ratios compared to the expected values from the *in silico* experiment outlined in panel (B). Divergence between expected and observed values occur because some fibers become too short to be consistently detected. (**D**) Computation of the median only over fibers present at all cut levels restores close agreement of DNAi-extracted values with expected ones.

Model performance was evaluated by monitoring changes in fiber-level ratio measurements, providing insight into the ensemble’s tolerance to real-world imaging. Figure 8A summarizes the results on a representative high-resolution image containing ∼300 fibers (obtained from DNAi, without perturbation). We compared four configurations: a single model, an ensemble, and an ensemble augmented with TTA ± error detection module.

While all configurations exhibit stability under mild perturbations, we observe a marked decline in the number of fibers detected under severe degradation. Notably, the ‘Ensemble’ configuration maintains consistent ratio measurements despite the reduced fiber count, indicating resilience in preserving biologically relevant metrics even when detection sensitivity is compromised. Quantitatively, from the original images to the most blurred ones ($\sigma = 12$), we observe variations in the measured median ratio of 2.92%, 2.10%, 1.76%, and 2.59% for the single model, the Ensemble, the Ensemble with TTA, and the Ensemble with TTA and error detection respectively. Under noise degradation, the variations are similar, at 3.11%, 1.16%, 1.43%, and 1.43%. These values should be interpreted together with detection performance: under blurring, the number of detected fibers drops substantially, by 48.4%, 59.2%, 63.4%, and 65.5% for the three configurations. In contrast, additive noise has a milder effect, with fiber detection decreasing by 24.3%, 29.3%, 39.3%, and 29.0%. While these drops in detection may appear substantial, they underscore the robustness of all configurations in preserving the central tendency of the measurements. Overall, these results demonstrate that DNAi’s ratio measurements remain stable under substantial image degradation, with median ratio variations below 3.2% even when more than half of the fibers are no longer detected. Ensemble configurations offer a slight advantage in measurement stability, while the error detection module preserves measurement accuracy despite its more aggressive filtering. Importantly, the consistency of the median ratio across degradation levels suggests that segmentation failures are not biased toward a particular subpopulation of fibers, but rather affect fibers uniformly across the ratio distribution, a desirable property for a quantitative analysis tool.

### Simulation framework for algorithmic bias and ratio control

We aimed to evaluate the algorithm’s performance on fibers with a wide range of nucleotide analog ratios, in order to highlight its ability to generalize across a controlled spectrum. To this end, we designed an *in silico* experiment that allows precise control over the ratio of fibers present in a slide, by artificially modifying fiber ratios in real images. Starting from an initial segmentation of the fibers, we shortened the skeleton of the segmented mask for the first analog, the second, or both. To propagate this modification onto the image, we generated a mask that encompasses the targeted cut region. Within this masked region, we applied a simple inpainting technique: each pixel was sampled from the area surrounding the fiber. Our method requires only two input parameters: the targeted analog (red, green, or both), and the fraction of its length to be removed. Figure 8B illustrates the outcome of this technique on the same fiber for different parameter values.

Given an initial ratio $\alpha$ and cut fractions per analog $C_r$ and $C_g$ (for red and green respectively), the measured ratio evolves as:


(1) \begin{eqnarray*}
f(C_r, C_g) = \alpha \cdot \frac{1-C_g}{1-C_r}
\end{eqnarray*}

The outcomes of the *in silico* experiments (Fig. 8C and D) closely follow theoretical expectations. The measured ratios evolve in line with the predicted values across all tested configurations. This strong agreement demonstrates that the model reliably segments fibers regardless of their individual lengths or ratios. Importantly, even when fibers are systematically shortened from one or both sides, the algorithm consistently recovers the correct population-level ratio. These results confirm the model’s capacity to generalize and maintain accuracy across a broad spectrum of fiber compositions.

### Detection of biologically relevant differences in IdU/CldU ratio

Blockage of DNA polymerase causes replication fork reversal[32]. During this process, the newly synthesized DNA strands anneal together, forming a four-way structure commonly referred to as a ‘chicken foot’. This exposes a double-stranded DNA end that is susceptible to nucleolytic degradation. So-called ‘fork protection’ factors, including BRCA1, BRCA2, and 53BP1, safeguard against this degradation thereby preserving nascent DNA integrity [33–37].

To evaluate the biological relevance and sensitivity of DNAi in detecting replication stress phenotypes such as nascent DNA degradation caused by lack of BRCA2, we compared fiber-level ratio distributions in the paired ovarian cancer cell lines PEO1 and PEO4. PEO1 cells are BRCA2-defective and exhibit impaired nascent DNA stability at stalled replication forks (i.e. fork protection defect), whereas PEO4 cells represent a BRCA2-reverted derivative with restored fork stability [35,38]. We also compared ratio distributions between control conditions (siNT) and siRNA-mediated gene-silencing targeting either BRCA1, BRCA2, or 53BP1. Cells were exposed to nucleoside analogs as described before ($30 \,\mathrm{min}$ CldU, wash, $30 \,\mathrm{min}$ IdU), followed by a 4 h treatment with HU to block replication fork progression. Nucleolytic degradation of the nascent DNA containing the second analog (IdU) during HU-induced fork stalling in cells lacking BRCA1/2 or 53BP1 is expected to cause a reduction in the IdU/CldU ratio compared to control cells.

**Figure 9.**
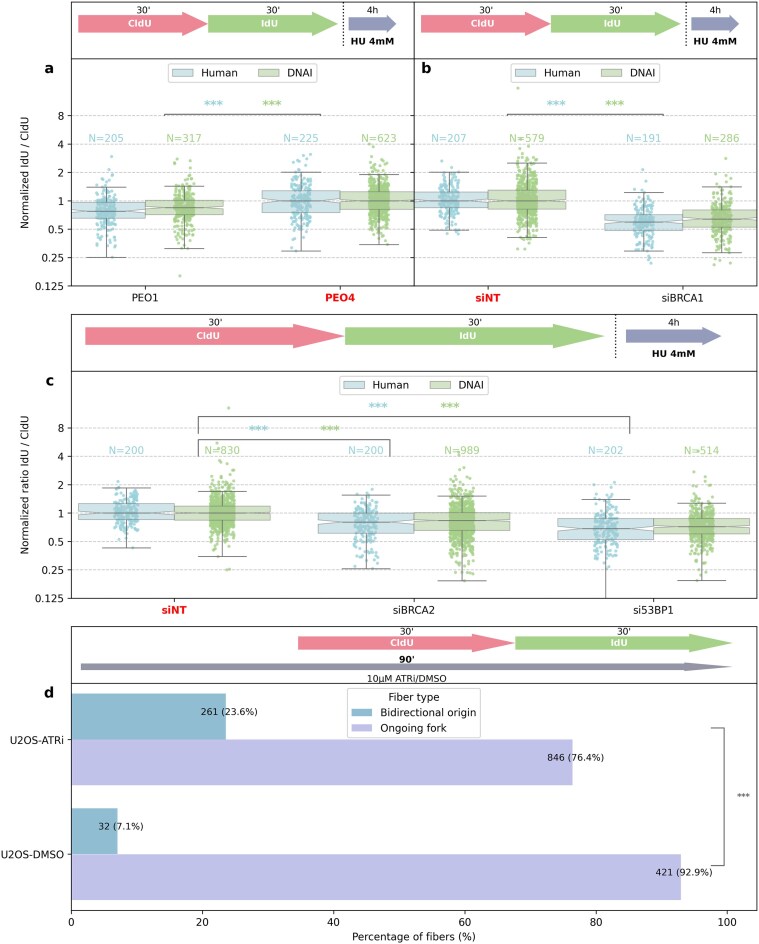
**(A-C**) DNA fiber analysis of nascent strand degradation following sequential CldU ($30 \,\mathrm{min}$) and IdU ($30 \,\mathrm{min}$) labeling and subsequent treatment with $4 \,\mathrm{m}\mathrm{M}$HU for 4 h to induce fork stalling. **(A)** Experiments done in PEO1 and PEO4 cells. (**B, C**) Experiments done in U2OS cells treated with the indicated siRNA prior to CldU/IdU labeling. Loss of fork protection factors (BRCA1, BRCA2, or 53BP1) causes the expected reduction in the IdU/CldU track length ratio relative to control. Conditions highlighted in red correspond to the reference (siNT or PEO4) whose median ratio is used to normalize all other values, independently for each grader (human or DNAi). Ratio distributions were compared to the reference using a Mann–Whitney U test, with significance indicated per grader (***P-value <.001). (**D**) Quantification of replication structures in U2OS cells treated with ATRi ($10 \,\mathrm{\mu }\mathrm{M}$) or DMSO as indicated, followed by sequential CldU/IdU labeling. The percentage of bidirectional origins and ongoing forks is shown. Fiber type distributions were compared using a chi-square test (***P-value <.001). Throughout the figure, the labeling protocol is illustrated schematically in each panel.

Figure 9A–C illustrate the distribution of ratio for each condition. As expected, we observed a statistically significant shift in the distribution in cells lacking BRCA1, BRCA2, or 53BP1, consistent with known effects on nascent DNA stability at stalled forks, in both the manual and DNAi measurements. These findings demonstrate that DNAi not only replicates the trends observed in expert annotations but also reliably captures expected changes in fiber ratios between biological conditions. The consistency of statistical significance across both annotation modalities underscores the robustness of the model.

#### ATR treatment

To assess whether DNAi can capture changes in DNA replication origin activity, we quantified replication structures in U2OS cells treated with ATR inhibitor (ATRi). As shown in Fig. 9D, ATRi increased the proportion of bidirectional origins relative to DMSO control, as previously reported [39–43]. Thus, beyond measurement of DNA fiber segments, DNAi can be used to accurately quantify the proportion of fiber structures (origin versus ongoing forks), thereby revealing expected shifts in replication patterns.

#### S1 nuclease

In addition to fork degradation, replication stress can result in the formation of ssDNA gaps behind replication forks [44, 45]. Mechanistically, ssDNA gaps can arise when DNA synthesis continues discontinuously after the replisome encounters stress, for example through PRIMPOL-dependent repriming, leaving stretches of unreplicated template behind the fork [46–48]. In contrast to fork degradation (which primarily indicates loss of the nascent label during prolonged stalling), gap formation is detected by a differential sensitivity of newly synthesized tracks to ssDNA-specific S1 nuclease-mediated cleavage [20, 49]. To broaden the range of experimental methodologies to which DNAi can be applied, we incorporated S1 nuclease assays into our training and analysis data set.

**Figure 10.**
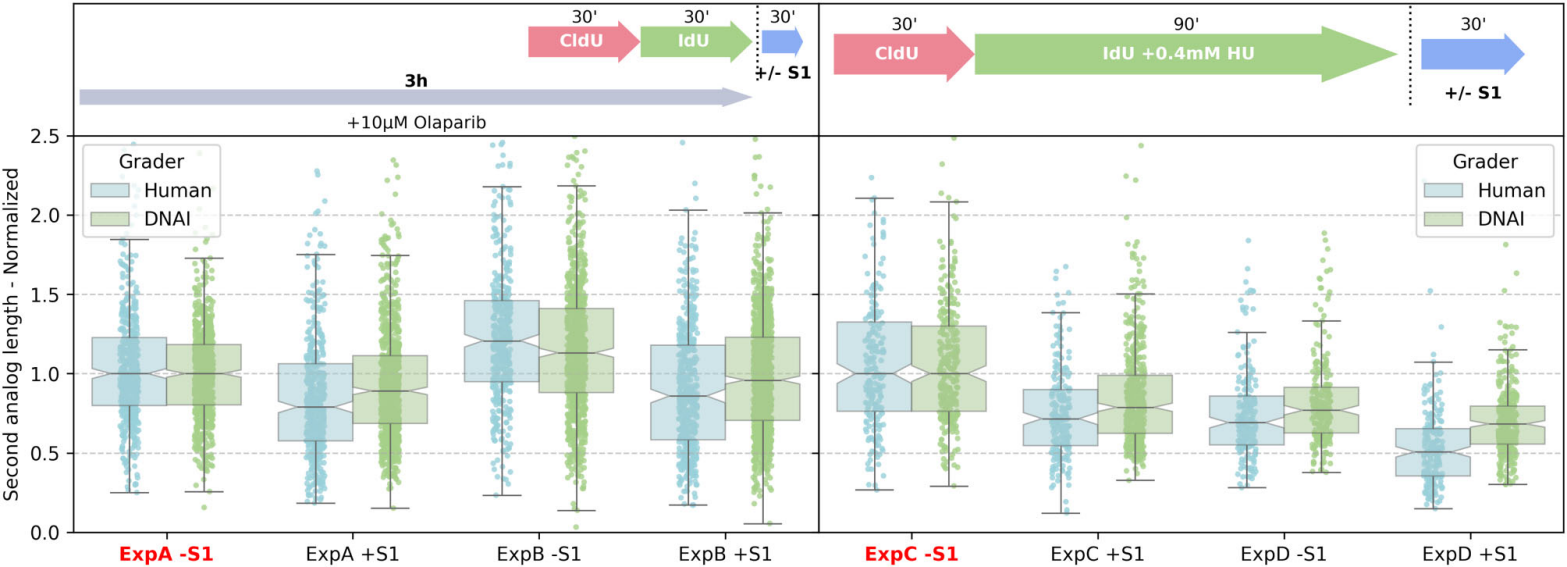
S1 nuclease DNA fiber assays showing replication-associated ssDNA gap formation following olaparib or low-dose HU treatment in U2OS cells. Schematic representation and quantification of normalized second-analog (IdU) track lengths in different conditions after treatment with $10 \,\mathrm{\mu }\mathrm{M}$ olaparib (left) or $0.4 \,\mathrm{m}\mathrm{M}$ HU during IdU labeling (right), followed by $\pm$ S1 nuclease digestion. IdU track lengths are normalized, independently for each grader (human or DNAi), by the median value of the reference condition (highlighted in red).

We performed DNA fiber assays in which nuclei were treated with S1 nuclease (or buffer control) prior to fiber spreading, and segment lengths were compared between ±S1 conditions. Because S1 nuclease converts ssDNA gaps within labeled nascent DNA into strand breaks, gap-containing fibers exhibit track shortening after S1 treatment, providing a quantitative proxy for post-replicative gap prevalence. The results indicate an overall excellent agreement between DNAi and human graders in this application (Fig. 10).

### Generalization to other acquisition device

**Figure 11.**
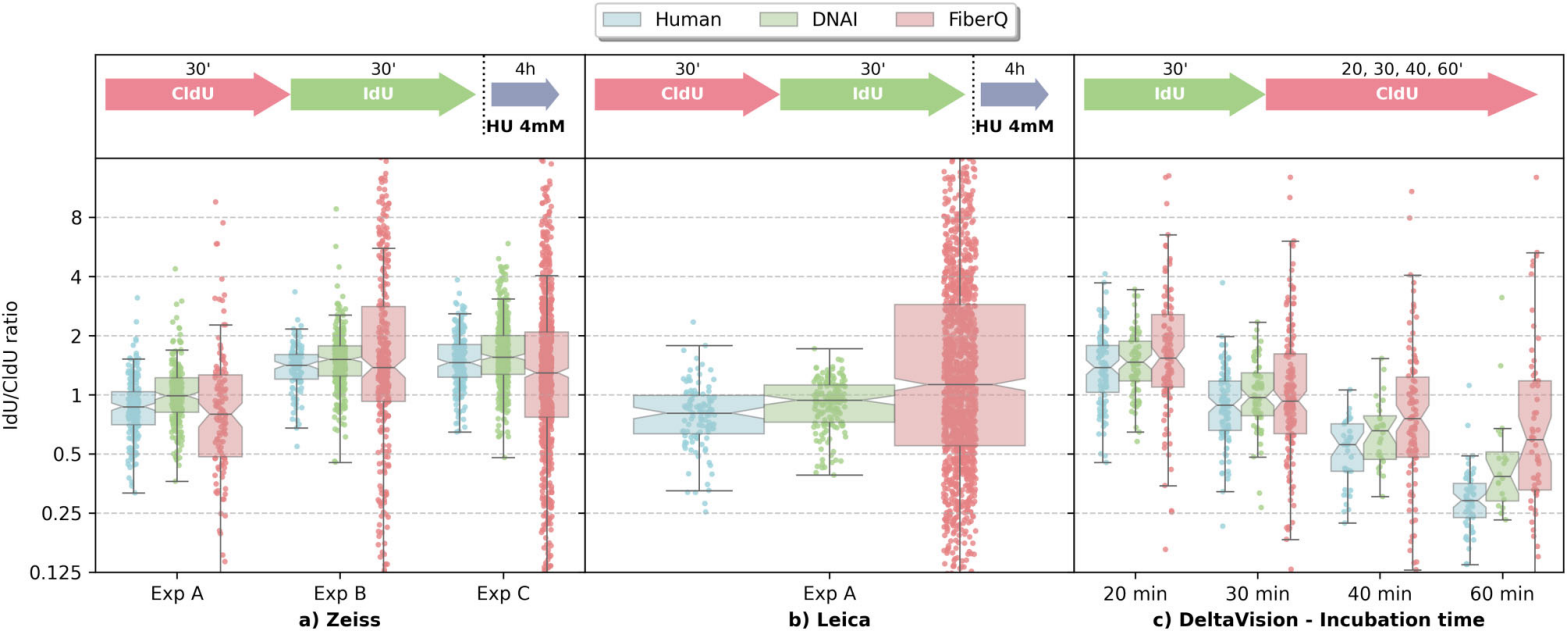
Generalization of DNAi to out of domain acquisition devices and comparison with FiberQ. **(A, B)** Fiber track length ratios measured by DNAi, FiberQ, and human annotators on images acquired with DeltaVision and Leica microscopes, neither of which were used during model training (Zeiss). **(C)** Time-course experiment in which the incubation order of the analogs is reversed (IdU first), with increasing CldU incorporation time. The expected decrease in the IdU/CldU ratio over time is consistently captured by DNAi, FiberQ, and the human grader.

**Figure 12.**
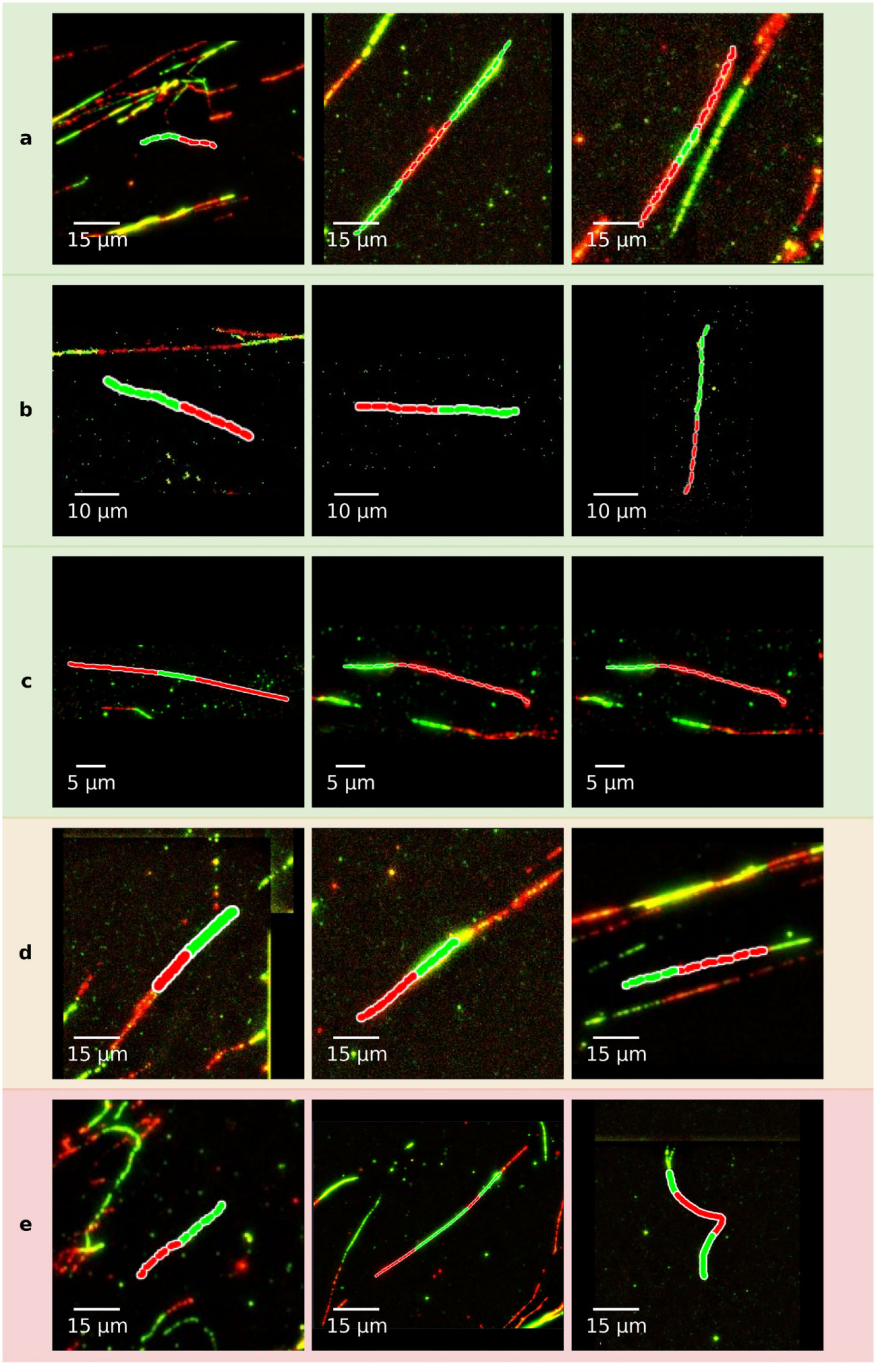
Qualitative examples of DNAi segmentation across different microscope platforms. (**A**–**C**) Segmentation on Zeiss (**A**), Leica (**B**), and DeltaVision (**C**) images. (**D**) Examples of mis-segmentation on Zeiss images correctly identified and filtered by the error detection model. (**E**) Examples of mis-segmentation not detected by the error detection model.

Images used for model training and for the tests described in the preceding figures were acquired on a Zeiss Axio Imager Z2 microscope (see the ‘Materials and methods’ section). We evaluated its performance on images acquired with a microscope from other manufacturer (DeltaVision, based on an Olympus platform, as well as a Leica microscope; see materials and methods). Contrast, pixel size and overall signal-to-noise appear to differ between images acquired using DeltaVision, Leica or Zeiss systems, which make the following experiments a good measurement of the robustness of the model to out-of-domain inference. As shown in Fig. 11, the data indicate that DNAi reliably captures relative trends in fiber ratios across experiments, even if minor shifts occur compared to the human counting that we did not observe at this scale on Zeiss images. Figure 12 illustrates representative examples of accurate segmentation across the above microscope platforms, as well as failures, both those correctly identified by the error detection module and those that went undetected and remain in the final output.

### Comparison with FiberQ

**Figure 13.**
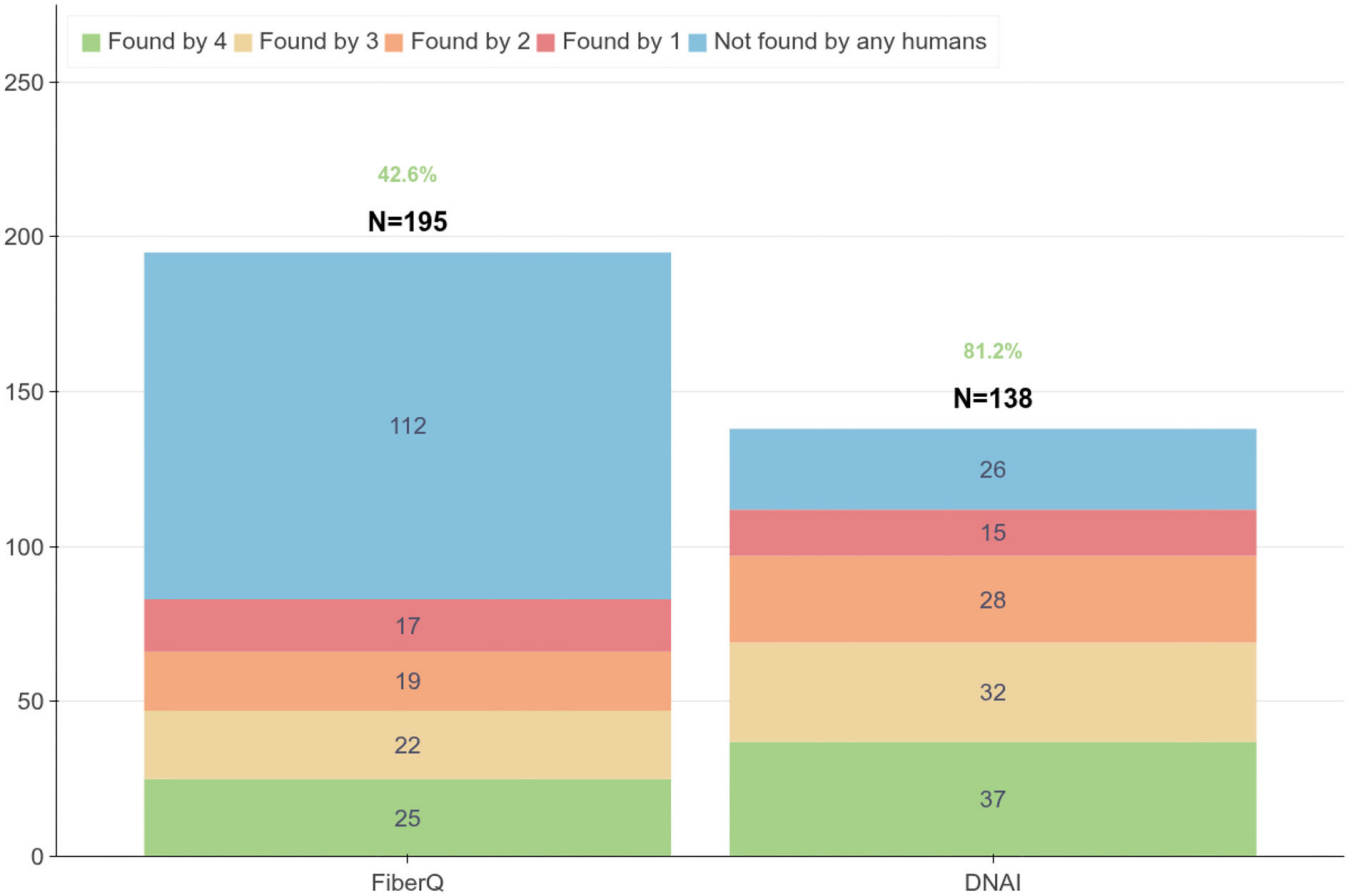
Stacked bar plot showing, for each detection method, the proportion of fibers also identified by one, two, or three human observers. FiberQ detects a large number of fibers not confirmed by any human grader, whereas DNAi maintains substantially higher agreement. These images were taken from the validation dataset (obtained using a Zeiss Axio Imager Z2 microscope).

We compared DNAi to FiberQ [21], another publicly available algorithm based on traditional computer vision techniques that was previously developed by our group. FiberQ was primarily optimized on images acquired with a DeltaVision microscope, whereas DNAi was trained exclusively on Zeiss microscopy data. To ensure a fair evaluation, we compared both systems on images from three microscopy platforms (Fig. 11). Both FiberQ and DNAi recover a median ratio hierarchy consistent with the human grader across all experimental conditions, although DNAi produces estimates that are generally closer to the human reference. We note, however, that FiberQ yields a substantially larger number of segmented fibers—many of which do not correspond to genuine structures, i.e. false positives. On the 20 images from our inter-grader dataset, 57.4% of the fibers detected by FiberQ were not identified by any human observer, compared to only 18.8% for DNAi (Fig. 13). In other words, nearly two thirds of FiberQ’s detections are likely false positives. Beyond accuracy, execution time differs substantially between the two tools. DNAi leverages GPU acceleration for both segmentation and feature extraction, resulting in ∼$10\times$ faster processing than FiberQ, which relies on sequential CPU-based operations. This speed advantage becomes particularly relevant when processing large batches of images.

### Rapid whole-slide analysis eliminates spatial sampling bias

**Figure 14.**
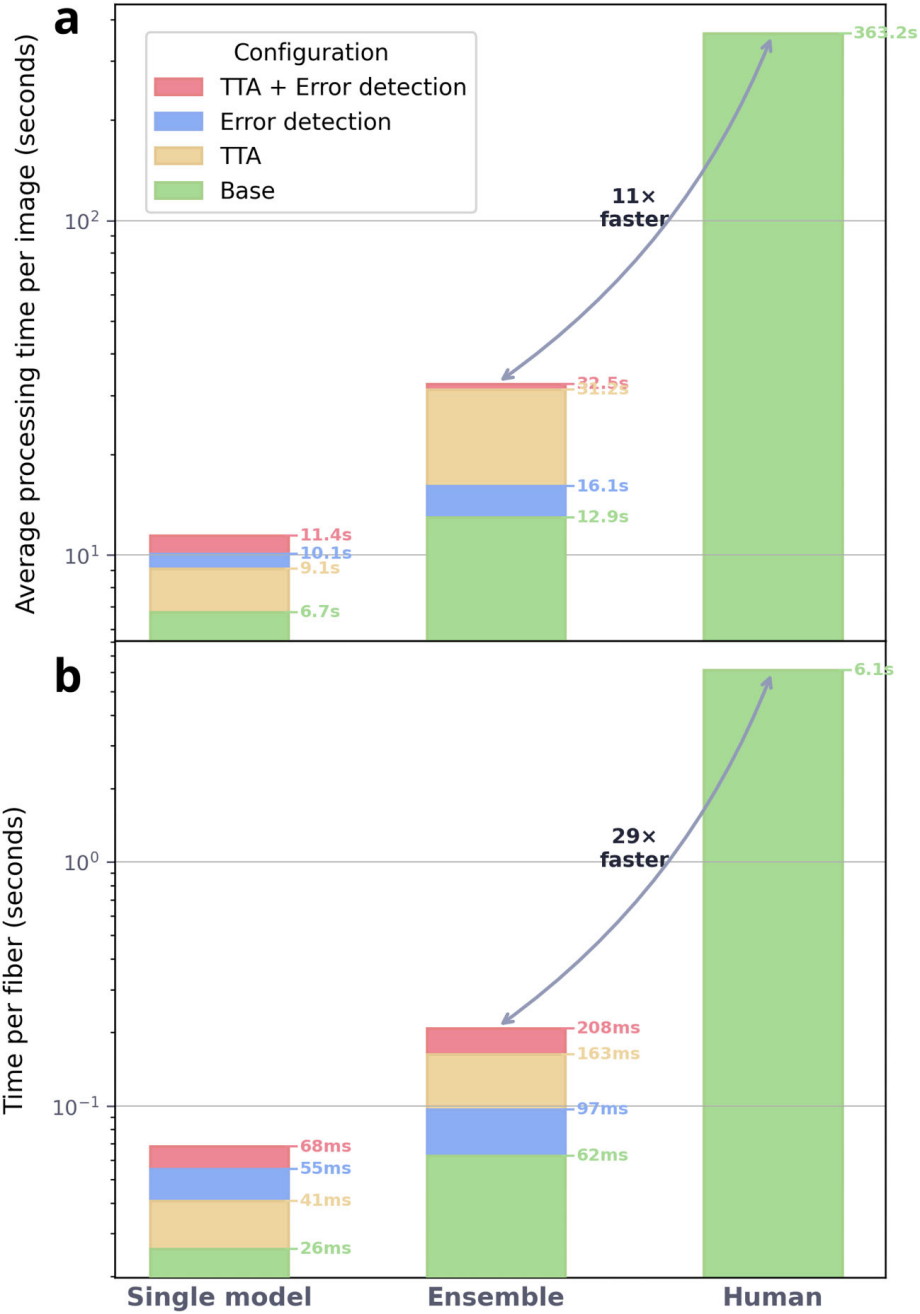
Processing time comparison between DNAi configurations and manual annotation, measured on eight whole-slide images ranging from $6975 \times 9712$ to $8467\times 23 \ 212$ pixels. **(A)** Average processing time per image. **(B)** Time per fiber. In both panels, bars are overlaid from tallest to shortest to show the incremental cost of each optional module. Even in its most computationally expensive configuration (ensemble with TTA and error detection), DNAi remains ∼$29\times$ faster per fiber, and $11\times$ faster per image than a trained human grader.

**Figure 15.**
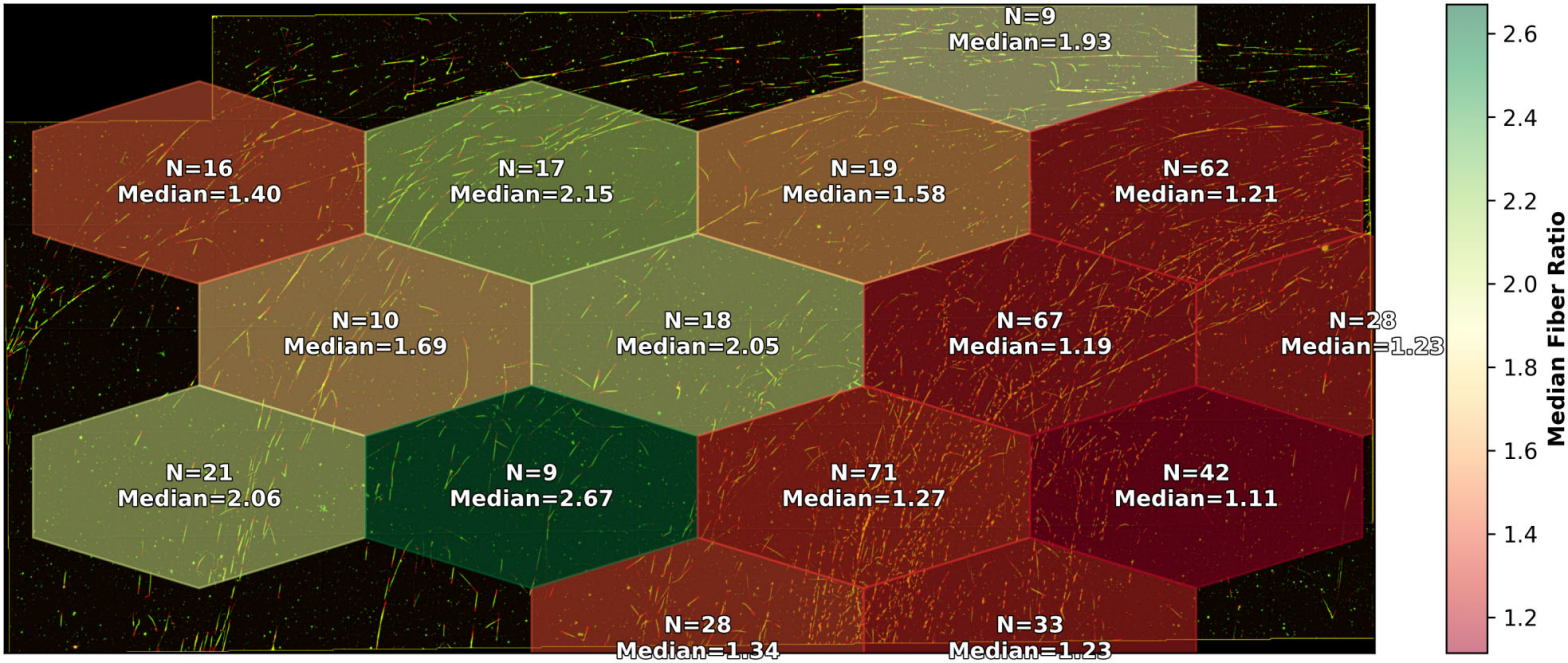
Spatial heterogeneity of fiber ratios within a single slide (several stitched images). Each tile shows the median IdU/CldU track length ratio computed over all fibers detected in that region.

A key advantage of automated analysis is its ability to process an entire slide without spatial bias. DNAi segments and measures a full stitched image in ∼10 s (for a $12 \ 000 \times 12 \ 000$ image), which is on average roughly $29\times$ faster per fiber than a trained human grader (a detailed analysis is provided in figure 14, though these numbers are hardware-dependent). In practice, human graders typically measure only the first 100–200 fibers they encounter, usually concentrated in a limited region of the slide. This introduces a spatial sampling bias: the reported ratios reflect only the local area where the observer began measuring, rather than the slide as a whole. To investigate whether this bias is meaningful, we examined the spatial distribution of fiber ratios across entire slides. We partitioned each image into nonoverlapping tiles and computed the median ratio per tile. The resulting ratio maps reveal substantial spatial heterogeneity: median ratios can vary by a significant factor across a single slide or image (figure 15). Because DNAi exhaustively processes every fiber regardless of its position, it provides a spatially unbiased estimate of the true ratio distribution. This whole-slide coverage not only reduces measurement variance but may also reveal biologically relevant effects that would otherwise go undetected by conventional manual scoring. These data also indicate that examining as much of each DNA fiber slide as possible is critical for accurately assessing track lengths from a given biological condition.

### Graphical user interface

To ensure usability, the entire pipeline is integrated into a graphical user interface (GUI) aimed at nontechnical users. The interface allows configuration of all analytical parameters and supports both single-image and batch processing. Visualization tools include an interactive display of original and segmented images, fiber bounding boxes, and a tabular view of individual fiber metrics. Users can manually exclude fibers from the analysis and generate population-level statistics using built-in calibration and regression features. To ensure cross-platform compatibility, the UI was developed as a web application running locally, allowing its use in any modern web browser.

In summary, the software is composed of the following configurable modules:

- Flexible image loading and preprocessing, including decoding of .cvi, .dv, .tiff, .jpeg, or .png formats. Preprocessing is configurable, allowing specification of pixel size, and analogs channels ordering. Users can load dual-channel images directly or load each channel separately.
- A segmentation module offering several pre-trained architectures with options for ensemble inference and/or TTA.
- An error-detection module that can filter false positives from the segmentation model with adjustable aggressiveness.

The viewer supports multiple visualization modes (bounding boxes, plain strokes, or animated strokes) and allows users to modify the status of a fiber (valid or invalid) with a simple click. Image-level statistics can be exported as a .csv file directly from the table view, which also enables detailed inspection of individual fibers. The GUI also allows users to adjust the prediction threshold applied to the detection model, which estimates the probability that each candidate fiber is a false positive. The default threshold is set at 0.5. Lowering this value applies a more stringent filter, rejecting a greater number of candidate fibers and thus reducing false positives at the potential cost of discarding valid structures. In addition, the GUI includes a batch analysis tab that sequentially processes all loaded files. The results can be exported as a.csv file or displayed as a swarm plot, with filenames automatically used to group images by experimental condition.

## Discussion

DNAi is a suite of software tools that can be used either via scripts or a graphical interface. It is intended as an open-source software that is easy to use and is expected to significantly improve the productivity of biologists performing DNA fiber assays. Our study demonstrates the technical feasibility of designing an AI for automatic segmentation of DNA fibers. Importantly, advances in microscopy enable the generation of increasingly large datasets from DNA fibers assays. We anticipate that DNAi and similar computer vision software will facilitate efficient analysis of these datasets, replacing manual counting of a limited number of fibers with more accurate, extensive, and reproducible analyses. We note that several methods have recently been developed to characterize DNA replication dynamics at the single-cell and single-molecule levels. Among these approaches, nanopore sequencing methodologies have been shown to permit unbiased quantification of replication fork progression at single nucleotide resolution [50, 51]. Other methods include microfluidics-based genome-wide optical replication mapping [52], super-resolution microscopy of nucleotide incorporation sites within nuclei (3D-SPARK) [53], and single-cell sequencing of nucleoside analog-labeled DNA [54]. Although these techniques enable highly detailed genomic and molecular analyses of DNA replication, they require specialized instrumentation and costly reagents, and the level of detail they provide is not always necessary to address fundamental questions related to replication fork progression and nascent DNA stability. Consequently, DNA spreading-based experimental strategies, which are inexpensive and rely on readily available widefield microscopes, are likely to remain widely used for many years. Nevertheless, higher-resolution approaches are expected to gain broader adoption in the coming years as costs decrease and technical barriers are reduced.

Our software not only expedites the quantification of DNA spreading experiments but also leads, in general, to a vastly increased number of detected DNA fibers per image. This is expected to improve the interpretation of experimental results: indeed, in our experience DNA spreading can lack uniformity in quality of labeled segments from one microscopy field to another. This can lead to biased results when experimenters only count a limited number of fibers (usually around *n* = 200) from a few microscopy fields. In addition, our previous data [21] and those presented here (Fig. 4) clearly demonstrate that the selection of fibers to be measured varies significantly between human observers, further biasing image interpretation. While our deep learning model is likely to introduce biases since it was trained on manually segmented images, it applies these biases uniformly across experimental conditions, thereby increasing reliability and reproducibility.

### Limitations

Despite its overall robustness, DNAi has several limitations that must be acknowledged. As mentioned before, the models were trained on a relatively constrained dataset, primarily composed of images resulting from labeling schemes consisting of two nucleotide analog pulses, which were acquired from a single microscope (Zeiss system), and were annotated by a limited number of experts. This introduces potential biases and limits generalizability. To mitigate this, we incorporated variability in the training set by varying experimental conditions, applied data augmentation, and used ensemble inference strategies. Moreover, we selected datasets which presented an approximately equal number of experiments whose median IdU/CldU ratios were either smaller, equal, or larger than 1, and containing fibers with ratios and length values significantly deviating from the median. In our hands, DNAi is able to detect a variety of DNA fiber length and analog ratios reflecting typical DNA spreading labeling schemes and results, and is not biased toward any type of ratio or DNA fiber length. Nevertheless, users should remain vigilant and verify results when using DNAi, especially when analyzing images originating from labeling schemes or experimental protocols that significantly deviate from those used to train our model.

Our model was trained and validated using the DNA spreading technique with a two nucleotide analog labeling scheme, i.e. resulting in two-color IF images. The software does not currently detect single color DNA fibers. Because of this, other deep learning models would likely need to be trained to robustly segment and quantify DNA fibers composed only of single label segments. We also note that while DNA combing and DNA spreading are conceptually related, DNA fibers resulting from the use of these methods differ noticeably with regards to length, shape, and continuity. While we have not tried to optimize DNAi for DNA combing applications, dedicated deep learning models will probably need to be trained specifically for this application.

While our data and validations indicate that DNAi is robust to a wide variety of imaging conditions, visual examination reveals that segmentation errors persist in the DNAi output, particularly when using images that are of lower quality or displaying densely packed fibers. While these errors affect individual measurements, analyses presented in the current work show that at the population level, their limited frequency limits their impact on distribution metrics. To address over-detection, which we observed across several models, we added an optional secondary error-detection module that considers both segmentation and local image context. This module improves alignment with human annotations in many cases but can reject fibers from imaging sources outside the training distribution. For example, we have observed empirically that images obtained using a DeltaVision microscope tend to result in a higher rejection rate than those generated using a Zeiss Z2 system. For this reason, we provided the option to disable this step via the graphical interface, which can improve segmentation with certain sets of images. This filtering model, while useful for removing obvious false positives, suffers from the same interpretability limitations as most discriminative neural networks. It may reject valid fibers without obvious justification, especially under conditions of image degradation.

These observations lead us to advise using the software through its graphical interface, which allows users to visually confirm that the model’s output aligns with human expectations. The GUI also exposes a range of adjustable parameters (e.g. pixel size, marker indexing order, model selection, detection sensitivity), enabling users to tailor the pipeline to their specific experimental setup. Visualization modules at the image, fiber, and distribution levels further support manual validation and correction.

In summary, our work demonstrates the feasibility of training deep learning models for DNA fiber quantification tasks. We anticipate that this and future software solutions will improve throughput and diminish inter-user variability and biases in the interpretation of such experiments, thereby improving the robustness and reliability of the results.

## Funding

Université de Montréal-Etudes supérieures et postdoctorales (ESP), WOCO foundation, Line Chevrette foundation, Natural Sciences and Engineering Research Council of Canada (NSERC) [RGPIN-2019-05082, RGPIN-2021-03330, RGPIN-2025-04612]. Funding to pay the Open Access publication charges for this article was provided by a NSERC discovery grant. 

## Data Availability

The data underlying this article (images and the corresponding masks used to train the models) are available at the following link: https://doi.org/10.5281/zenodo.18868353. The complete code for training and using DNAi is available in the following github repository: https://github.com/ClementPla/DNAi (archived at https://doi.org/10.5281/zenodo.19237854). Models weights are automatically downloaded on usage from a repository on HuggingFace.
